# Tuning lipid accumulation and fitness of motile algae via hydrodynamic cues

**DOI:** 10.3389/fbioe.2025.1722499

**Published:** 2026-01-05

**Authors:** Narges Kakavand, Anupam Sengupta

**Affiliations:** 1 Physics of Living Matter, Department of Physics and Materials Science, University of Luxembourg, Luxembourg City, Luxembourg; 2 Institute for Advanced Studies (IAS), University of Luxembourg, Esch-sur-Alzette, Luxembourg

**Keywords:** motile microalgae, Heterosigma akashiwo, hydrodynamic cues, lipid accumulation, biomass production, photophysiology, growth kinetics, algal biofuels

## Abstract

Achieving enhanced lipid yield without compromising biomass is a central challenge for sustainable algal biofuel production. While temperature, nutrients, and light can induce lipid accumulation, they often reduce overall fitness, offsetting net gains. By contrast, hydrodynamic cues remain underexplored, particularly in the context of motile algae and their physiological response in terms of fitness and lipid production. Here, we investigate *Heterosigma akashiwo*, a well-known motile phytoplankton species, exposed to controlled hydrodynamic cues at two physiological stages: immediately after inoculation (lag phase) and during the mid-exponential growth phase. We quantify intracellular lipid accumulation, growth kinetics, and photophysiology, and compare these parameters between two different strains of *H. akashiwo*. Early induction of hydrodynamic cues (during the lag phase) increased average cytoplasmic lipid accumulation by nearly 300% at the single-cell level, without adverse effects on fitness and biomass production. Growth rate accelerated while photophysiological performance was preserved. In contrast, delayed induction (exponential phase) yielded only marginal lipid enhancement and reduced biomass and photosynthetic efficiency. At the strain level, these trends were consistent, while we note strain-specific differences in the extent of the response. These results identify the onset timing of hydrodynamic cues as a tunable parameter to enhance lipogenesis while preserving physiological fitness, suggesting a simple and potentially scalable route to improve lipid production using motile microalgae.

## 1 Introduction

Biofuels, renewable and environmentally friendly alternatives to fossil fuels (Chen et al., 2021), have received renewed attention during the past decade in light of the exacerbated global warming (Shindell and Smith, 2019; Vertès et al., 2006; Brahma et al., 2022). Although awareness and demand for green bio-based alternatives to fossil fuels is at a peak, their energy efficiency and economic viability remain a moot point, preventing universal acceptance of biofuels (Hoang et al., 2023). Relatively lower energy yields (Sharma et al., 2018), particularly in terrestrial plants which were the primary feedstock for biofuel production, have been a key hurdle. In contrast, microalgae have shown promise as the next-generation of biofuel feedstock, thanks to their superior energy density, efficient growth, and ultimately higher yield as a sustainable source of energy (Mat Aron et al., 2020; Thanigaivel et al., 2022; Zhu et al., 2022; Hoang et al., 2023). Beyond the biochemical advantages, microalgae offer operational benefits such as simplified cultivation, economic viability, and reduced environmental footprint in generation, further consolidating their preferred status over traditional plant-based feedstocks (Thanigaivel et al., 2022).

Microalgae, equipped with chlorophyll, perform oxygenic photosynthesis—using light energy to fix atmospheric carbon dioxide into organic matter and releasing molecular oxygen derived from water. While microalgae hold exceptional promise as a feedstock for biofuel, sustainable production still faces obstacles due to the disproportionate energy requirements for biofuel production and extraction from algal feedstock (Mat Aron et al., 2020; Khan et al., 2018; Choi and Lee, 2022; Ngatcha et al., 2022). At the scale of a single cell, biofuels are produced in the form of cytoplasmic lipid droplets (Sengupta et al., 2022; Hoang et al., 2023), often under nutrient limitation, or in response to altered light regimes and mechanical perturbations that act as biophysical stressors. Physiological stress due to altering light intensities (Nzayisenga et al., 2020; Maltsev et al., 2021b; Chin et al., 2023), salinity (Atikij et al., 2019; Pancha et al., 2015; Salama et al., 2014), nutrient availability (nitrogen and phosphorus deficiencies) (Sengupta et al., 2022; Yang et al., 2018; Maltsev et al., 2021a), temperature (Gao et al., 2023; Morales-Sánchez et al., 2020), and mechanical compression (Cho and Shin, 2016) have been employed to drive algal lipid production. Stressors, while promoting intracellular lipid accumulation, inadvertently compromise photophysiology and biomass production, suppressing overall fitness (Teh et al., 2021; Nagappan et al., 2019). In agreement with fundamental studies on the interactions between microalgae and their dynamic environments (Carrara et al., 2021; Sengupta et al., 2017; 2022), the stressful conditions—often a requirement for algal biofuel production—lead to lower biomass, thus offsetting higher yields with reduced total biomass. Two-stage cultivation systems, employing an initial stage for microalgal growth followed by induction of biofuel-regulating conditions, offer a way around (Nagappan et al., 2019), but they come at a higher production cost. Consequently, achieving a cost-effective balance between promoting growth and enhancing lipid production, and understanding the factors underpinning this balance, remain central challenges in algal biofuel research (Jan et al., 2023).

Among the various microalgal species used in biofuel production, the majority are non-motile, chosen for their relatively higher lipid contents and predictable growth patterns, making them suitable for bioreactor cultivation (Hoang et al., 2023; Peng et al., 2020; Martinez Carvajal et al., 2024). More recently, motile species have emerged as promising candidates, including *Chlamydomonas reinhardtii* (Martinez Carvajal et al., 2024), *Dunaliella tertiolecta* (Patel et al., 2023), *Dunaliella primolecta* (You et al., 2020), *Tetraselmis suecica* (Reyimu and Özçimen, 2017), *Isochrysis galbana* (Sánchez-Bayo et al., 2020), and *Haematococcus pluvialis* (Hosseinia et al., 2020). In motile microalgae, environmental forcing—light, nutrients, gas composition—can be used to steer cellular allocation while sustaining productivity. Disruption of motility due to environmental factors can trigger physiological stress, which may be leveraged for lipid production (Sengupta et al., 2017; Carrara et al., 2021; Sengupta et al., 2022; Sengupta, 2023).

Within this context, raphidophytes offer distinct advantages for biofuel production, including rapid proliferation rates (Sengupta et al., 2017; Ghoshal et al., 2024) and the absence of a conventional cell wall, enabling direct lipid extraction without the additional energy costs associated with cell wall disruption (Kwok et al., 2023; Lum et al., 2022). Yet, they remain largely underexplored due to limited studies on their cultivation, lipid accumulation kinetics, and optimized extraction protocols (Fuentes-Grünewald et al., 2012; Gallardo-Rodríguez et al., 2020; Lin et al., 2020).

*Heterosigma akashiwo* (hereafter, *H. akashiwo*), a motile raphidophyte, has demonstrated consistent neutral-lipid accumulation under nutrient limitation, with strain-specific variation in lipogenesis rates (Sengupta et al., 2022). It also performs robustly under conditions relevant to industrial scale-up, such as growth on ${\text{CO}}_{2}$ /NO_x_-rich gas streams, while maintaining photosynthetic performance and favorable biomass composition (Stewart et al., 2015; Bianco et al., 2016; Healey et al., 2023). Temperature (Mehdizadeh Allaf and Trick, 2023; Allaf and Trick, 2024; Fuentes-Grünewald et al., 2012; Thangaraj and Sun, 2023), salinity (Mehdizadeh Allaf and Trick, 2023; Allaf and Trick, 2024; Salama et al., 2014), aeration (Lou et al., 2020; Fuentes-Grünewald et al., 2013), and mechanical stirring (Gallardo-Rodríguez et al., 2020) have all been shown to influence lipid production in *H. akashiwo*; however, several of these strategies incur fitness costs (Gallardo-Rodríguez et al., 2020; Fuentes-Grünewald et al., 2013). This trade-off motivates exploring physical drivers that might tune lipid allocation while preserving growth and photo-physiology.

Among such drivers, hydrodynamic forcing remains comparatively underexplored for *H. akashiwo*, even though it strongly shapes the behavior and physiology of motile phytoplankton (Sengupta et al., 2017). In *H. akashiwo* and related motile raphidophytes, small-scale turbulence can perturb swimming trajectories and migratory behavior (Sengupta et al., 2017; Sengupta, 2023), altering residence times within local light and nutrient microenvironments. At the same time, flow reorganizes the physicochemical setting by modulating light–dark exposure (the “flashing-light” effect) (Fernández et al., 1999; Sato et al., 2010; Abu-Ghosh et al., 2016; Huang et al., 2017; Saccardo et al., 2022; Chiarini and Quadrio, 2021), enhancing ${\text{CO}}_{2}$ delivery and ${\text{O}}_{2}$ stripping (Abu-Ghosh et al., 2016; Huang et al., 2017), and reducing self-shading (Sato et al., 2010; Abu-Ghosh et al., 2016; Saccardo et al., 2022). These behavioral and transport pathways can, in turn, reprogram cellular metabolism and redistribute carbon allocation, providing a mechanistic route from flow to lipid allocation. Nevertheless, a mechanistic account of how motile *H. akashiwo* cells allocate resources toward neutral-lipid storage in response to hydrodynamic forcing—and how the timing of that forcing along the growth curve governs the outcome—has not been established.

Motivated by pronounced intra-specific diversity in *H. akashiwo*—including documented genetic separation among strains and persistent differences in behavior and physiology across environments (Sengupta et al., 2022; Harvey et al., 2015)—we selected two genetically and behaviorally contrasting isolates, CCMP452 (Narragansett Bay, Rhode Island, USA (Bigelow Laboratory for Ocean Sciences, National Center for Marine Algae and Microbiota NCMA, 2025b) and CCMP3107 (Nowish Inlet, British Columbia, Canada (Bigelow Laboratory for Ocean Sciences, National Center for Marine Algae and Microbiota NCMA, 2025a), to determine how hydrodynamic cues—and, critically, their onset timing (at inoculation versus mid-exponential growth)—govern intracellular lipid allocation, population growth kinetics, and photo-physiological performance. While mixing is well established to enhance growth and metabolism (including lipid synthesis) in non-motile microalgae (Fernández et al., 1999; Sato et al., 2010; Abu-Ghosh et al., 2016; Huang et al., 2017), extrapolation to motile taxa is not straightforward: even modest shear can disrupt flagellar function and swimming behavior, depressing fitness at lower thresholds than in non-motile species (Sengupta et al., 2017; Thomas and Gibson, 1990; Gallardo-Rodríguez et al., 2020; Wang and Lan, 2018; Jaouen et al., 1999). Thus, although mixing-based enhancement has been proposed broadly, a mechanistic account for motile raphidophytes has remained unresolved. Here we deliberately probe a hydrodynamic regime that is stronger than typical mixed-layer conditions in nature and overlaps ranges previously reported as harmful for motile phytoplankton (Sengupta et al., 2017; Thomas and Gibson, 1990), enabling a rigorous test of how flow—and its timing along the growth curve—reprograms lipid allocation, growth, and photo-physiology in *H. akashiwo*.

## 2 Materials and methods

### 2.1 Cell culturing in static conditions

Two strains of *H. akashiwo*, CCMP452 and CCMP3107 (hereafter HA452 and HA3107), were cultured under sterile conditions in 50 mL glass tubes using f/2 (–Si) medium (Guillard, 1975). Artificial seawater (ASW) was prepared in-house by dissolving 36 g of sea salt per liter of Milli-Q water (final salinity 35–36 PSU; pH 8.0 ± 0.1). Macronutrients in f/2 (–Si) were at the standard final concentrations (no silicate): nitrate 882 μ M (as ${\text{NaNO}}_{3}$ ) and phosphate 36 μ M (as ${\text{NaH}}_{2}$ PO ${}_{4}\cdot$ H ${}_{2}$ O). Trace metals and vitamins were added at the canonical f/2 levels as in (Guillard, 1975).

All procedures were conducted under strict aseptic conditions inside a Class II laminar flow hood. The work area was sterilized with 70% ethanol before and after each session. Glass culture tubes and pipette tips were autoclaved prior to use, and all transfers were performed with sterile serological pipettes to minimize contamination. Operators wore sterile gloves and re-sanitized with ethanol during manipulations.

Three biological replicates per strain were propagated from mother cultures in exponential phase: 2 mL of well-mixed cell suspension was aseptically taken from the upper layer (top 0.5 cm) of the mother culture and inoculated into 25 mL fresh f/2 (–Si) medium under the hood. Each replicate was maintained in an individual tube to avoid cross-contamination.

For each strain, two sample sets were prepared (each set comprising three tubes derived from independent mother cultures). Cultures were incubated under a 14:10 h light:dark cycle at 22 °C with white LED illumination (peak λ≈535 nm) at an intensity of $1.35\;mW,cm^{-2}$ ( $\approx 60\;\mu mol\;photons\;m^{-2}\;s^{-1}$ ) during the light phase. Lights were on from 04:00 to 18:00 and off from 18:00 to 04:00. Unless otherwise specified, all experiments were conducted between 10:00 and 16:00. Each strain included three biological replicates, with multiple technical replicates as detailed elsewhere.

### 2.2 Cell culturing under hydrodynamic perturbations

Cell cultures were prepared following the protocol outlined above. Thereafter, two different hydrodynamic configurations were investigated: Scenario 1 with 0-h delay, wherein samples were exposed to hydrodynamic cues right after their inoculation in fresh media. The freshly inoculated cell cultures were placed on an orbital shaker housed within the same incubator as the control culture. The shaker was operated at a speed of 110 revolutions per minute (rpm), introducing a hydrodynamic stimulus as the sole variable relative to the control sample. The orbital shaker provided a mild swirling action to stir the culture medium, thereby generating a hydrodynamic perturbation within the cell culture.

**FIGURE 1:**
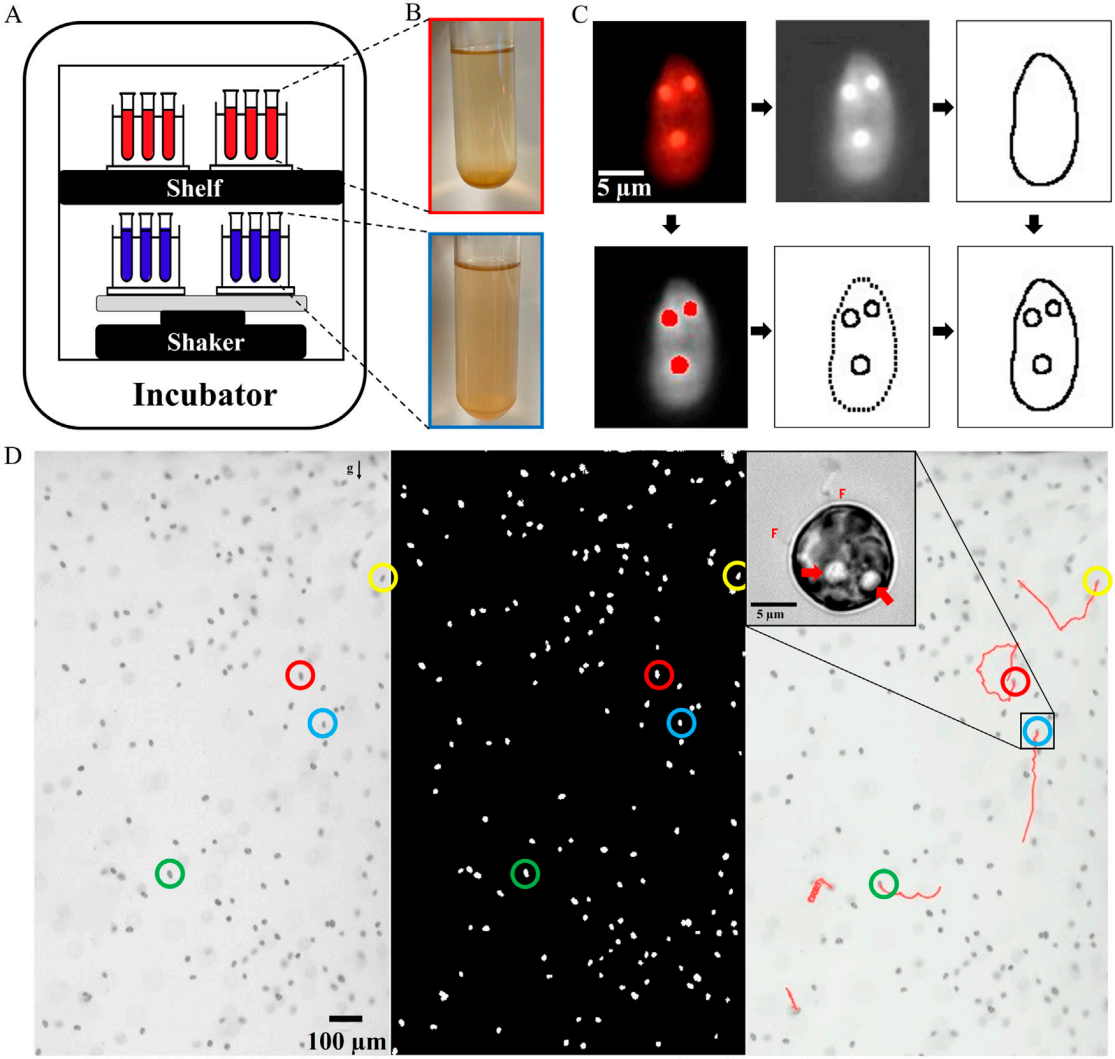
Experimental concept showing analysis pipeline for quantification of physiology and lipid generation. **(A)** The schematic illustrates our experimental concept: Two sets of cell cultures are housed within a temperature- and light-controlled incubator. The first set is maintained under static conditions (control population), while the second set is exposed to hydrodynamic perturbation on an orbital shaker. The blue and red colour scheme is used throughout the article to indicate the populations on the shaker (exposed to hydrodynamic cues) and the static (control) populations respectively. **(B)** Snapshot of the two representative culture tubes, photographed here 180 h after inoculation. **(C)** Quantitative analysis of the intracellular lipids and the cell morphology, obtained using thresholding algorithms to estimate the relative volume of lipid droplets compared to the total cell volume. **(D)** Visualizing swimming cells in a vertical millifluidic chamber allows quantification of the cell concentration and dynamics, with the direction of gravity indicated by the gravity vector ‘g’. The analyzed image (middle panel) displays bright spots representing cells after background subtraction. The right panel presents motile cells, with a few representative trajectories. The inset shows an *H. akashiwo* cell with its two flagella (indicated as F) and two lipid droplets appearing as bright spheres (indicated by two arrows), imaged at ×100 magnification.

For the second configuration, i.e., Scenario 2 with 120-h delay, the cell cultures underwent an initial static incubation period, similar to the control population. Once the cell cultures reached mid-exponential phase, they were subjected to the aforementioned hydrodynamic perturbations via the orbital shaker. This approach ensured that, aside from the introduced hydrodynamic cues, all samples experienced identical environmental factors (see Figure 1A), thereby facilitating a controlled comparison of the effects of hydrodynamic versus static growth conditions.

The orbital-shaker geometry and fluid parameters were defined following the empirical formulation of (Gallardo Rodríguez et al., 2009) for orbital mixing systems. The working cultures were maintained in 55 mL glass tubes (ROTH ROTILABO) with an inner diameter ( D ) of 23.0 mm, a working volume ( $V_{f}$ ) of 27 mL (25 mL of fresh f/2 (–Si) + 2 mL of cell suspension), and a liquid column height ( H ) of approximately 7.5 cm. The shaker had an orbit diameter ( $d_{o}$ ) of 20 mm. Using these parameters, together with seawater properties at 22 °C—density ( $\rho_{f}$ ) = 1,025 kg m^−3^ and dynamic viscosity ( $\mu_{f}$ ) = 1.1 mPa s—the hydrodynamic energy dissipation rate was calculated using the correlation

$$\varepsilon =\frac{1.94\;n^{3}D^{4}}{V_{f}^{2/3}\;{\left(\frac{\rho_{f}\;n\;D^{2}}{\mu_{f}}\right)}^{0.2}},$$ (1)

where n is the platform rotational speed, D the tube inner diameter, and $V_{f}$ , $\rho_{f}$ , and $\mu_{f}$ denote the working volume, fluid density, and dynamic viscosity, respectively. By substituting these values, with n = 110 rpm and seawater properties at 22 °C into Equation 1, we obtain:

$$\varepsilon \approx 9.54\times 10^{-4}\;Wkg^{-1},$$

representing the average rate of energy dissipation per unit fluid mass once steady-state swirling was reached.

The steady-state condition for the hydrodynamic regime was defined as follows: (i) the platform rotational speed n maintained at 110 rpm, stable within ± 1% according to the controller readout, for at least 5 min; (ii) a time-invariant swirling flow in the culture tube, evidenced by an unchanging meniscus trajectory over the same period; and (iii) the incubator temperature stabilized at 22 °C ( ± 0.2 °C), ensuring that $\rho_{f}$ and $\mu_{f}$ remained constant. Under these conditions, the parameters n , D , $V_{f}$ , $\rho_{f}$ , and $\mu_{f}$ were treated as time-independent, and ε was computed accordingly.

To verify the time required to reach steady state in this system, control experiments were conducted using a food dye solution matched in density to the cell cultures. The orbital shaker was operated under identical conditions, and the dye mixing dynamics were visually monitored. These tests revealed that homogeneous mixing—and thus a steady swirling pattern—was achieved in under 1 hour, a period negligible compared to the multi-week duration of the algal growth experiments. Using the above definitions and parameters, the steady-state hydrodynamic energy dissipation rate was approximately one order of magnitude above dissipation levels typical of wind-driven mixed-layer turbulence in the upper ocean (Sengupta et al., 2017).

Before selecting the standard operating condition, preliminary trials were conducted to evaluate the effects of different orbital speeds (50, 110, and 220 rpm) under identical culture geometry and volume. At 50 rpm, the hydrodynamic perturbation was insufficient to produce measurable differences from static controls in either growth or behavior. In contrast, 220 rpm induced visible stress responses, including rounded cell morphology and increased susceptibility to lysis following Nile∼Red staining (to label cytoplasmic lipid droplets), suggesting excessive shear. The intermediate speed of 110 rpm provided a reproducible hydrodynamic perturbation that clearly differentiated the outcomes from static controls without inducing morphological or physiological stress. To isolate the effect of perturbation timing while avoiding confounding effects from multiple shear regimes, all systematic experiments were therefore performed at 110 rpm. Throughout the 2-week experimental period, phase-contrast inspections confirmed that cells remained motile and morphologically intact under this condition in the 0-h delay scenario.

### 2.3 Variation of nutrient levels in microalgal cultures over time

To assess nutrient concentrations within the *H. akashiwo* culture, samples were collected at regular intervals throughout the growth period. The residual concentrations of nitrate and phosphate in the medium were quantified spectroscopically using a Spectroquant^®^ Prove 600 spectrophotometer, following the manufacturer’s protocols and reagent kits specifically designed for nitrate and phosphate analysis. Details of the protocol can be found in an earlier report (Sengupta et al., 2022).

### 2.4 Quantifying *Heterosigma akashiwo* growth kinetics

To accurately assess cell concentration over time, small aliquots were collected from the culture tubes and counted using a microscope to establish precise cell counts (Sengupta et al., 2017). This procedure was repeated for all experiments, spanning multiple biological and technical replicates.

At the time of inoculation ( t=0 ), the initial cell concentration was determined indirectly from the mother culture used for inoculation, quantified using a custom, in-house–fabricated millifluidic observation chamber made of PMMA (polymethyl methacrylate; not commercially sourced) with internal dimensions 3 mm × 2 mm × 0.56 mm (approximately 3.4 µL). The visualization setup followed our previous work and is described in (Sengupta et al., 2022).

Given that 2 mL of this suspension were transferred into a 27 mL working volume, the starting concentration in each culture tube was estimated as

$$C_{0}=N_{\text{mother}}\times \frac{2}{27}\;\;{\text{cells mL}}^{-1}.$$ (2)

We verified this independently by microscopy on separate culture tubes that were not used in the experiments. Direct on-tube sampling began at 24 h after inoculation to avoid disturbing cells during their initial acclimation to the fresh growth medium and environmental conditions. To ensure representative sampling, each culture tube was gently rolled two to three times before sampling to homogenize the suspension without introducing strong shear. The aliquots were consistently withdrawn from the mid-column along the central axis of the tube, avoiding both the surface and bottom regions. This procedure was applied uniformly to both static and orbital-shaken cultures: while the shaken cultures already exhibited a more homogeneous distribution (Figure 1B) due to continuous recirculation, the same gentle rolling and mid-column sampling protocol was applied across all samples to maintain procedural consistency.

The collected samples were then transferred into the same millifluidic PMMA chamber described above and imaged using the same stereomicroscope setup at 16 fps for 10 s to acquire dynamic image sequences. These image series were processed using MATLAB’s Computer Vision Toolbox (Version 9.10.0.1669831) to count cells in each frame and obtain the average concentration per sample.

To determine cell concentration, the average cell count across 160 frames (10 s × 16 fps) was divided by the chamber volume (3.4 µL) to yield a concentration in cells μ L ${}^{-1}$ , then multiplied by 1,000 to convert to cells mL ${}^{-1}$ . For samples with high cell densities (typically after 5–6 days), cultures were diluted prior to imaging, and the final concentration was back-calculated to account for the dilution factor.

The aggregated cell concentrations were plotted over time at intervals of 24, 48, 72, 96, 192, 264, 336, and 384 h, or until a plateau was reached, indicating the carrying capacity. At 0 h, the cell concentration was estimated using Equation 2.

The growth curves were then fitted to a logistic model to determine the doubling time and carrying capacity of each population. When plotted in logarithmic scale, the linear segments indicated the exponential growth phase, from which the doubling time ( $T_{d}$ ) and specific growth rate ( r ) were derived. The logistic growth equation was expressed as:

$$P\left(t\right)=\frac{K}{1+\left(\frac{K-P_{0}}{P_{0}}\right)e^{-rt}}$$ (3)

where K is the carrying capacity, $P_{0}$ the initial population, and r the specific growth rate. The doubling time was calculated as:

$$T_{d}=\frac{\ln\left(2\right)}{r}$$ (4)

Here, $T_{d}$ represents the time required for the population to double during the exponential phase, and K denotes the maximum population size sustainable under given resource and biochemical constraints.

Parameter estimation was performed via nonlinear regression fitting of the logistic model to the measured data (see Supplementary Figure S1). The resulting fits provided quantitative measures of population kinetics and long-term growth potential under both static and hydrodynamic perturbation conditions. Independent validation via flow cytometry and particle tracking at random time points confirmed that the MATLAB-based counts were consistent across methods (Supplementary Figure S2).

We define a growth index, $I_{G}$ , to quantify the relative change in population size for both the scenarios spanning the growth phases (upper inset in Figures 3A,D. The green plot indicates the 0-h case, while magenta curve plots the same for the 120-h delay case. The growth index, denoted by $I_{G}$ , is defined as:

$$I_{G}=\frac{K_{\text{Perturbed}}}{K_{\text{Static}}}$$ (5)

where $K_{\text{Static}}$ represents the cell concentration of the static cells at each time point, derived from the corresponding logistic model, and $K_{\text{Perturbed}}$ represents the cell concentration of the perturbed cells at each time point, also derived from their corresponding logistic model.

### 2.5 Flow cytometry

To ensure accurate and reproducible quantification of cell concentration, flow cytometry was used to validate and optimize the image-based counting procedure. Measurements were performed using an *Attune™NxT Acoustic Focusing Cytometer* (Thermo Fisher Scientific) operated in volumetric counting mode, which enables direct event-based quantification without reference beads. For this counting-only assay, the purpose was to determine cell number rather than physiological status; therefore, aliquots with estimated concentrations exceeding 10,000 cells (typically after 100–120 h post-inoculation) were briefly diluted in Milli-Q® water to improve optical clarity and prevent coincidence artifacts.

Phytoplankton cells (*Heterosigma akashiwo*) were identified by intrinsic chlorophyll autofluorescence excited at 488 nm and detected in the BL3-H channel. Sequential gating was applied to exclude debris and doublets: (i) fluorescence-based gating to isolate the autofluorescent population, followed by (ii) forward scatter (FSC) and side scatter (SSC) gating to assess cell size and internal complexity. Acquisition volumes and dilution factors were recorded to compute final cell concentrations (cells mL −1 ). These values were then compared against results from the image-based MATLAB counting scripts, confirming strong agreement with discrepancies below 10%.

### 2.6 Measuring lipid production using cell-scale microscopy

Intracellular lipid droplet (LD) biosynthesis and accumulation were quantified at predefined time points. Prior to sampling, cultures were gently mixed to ensure a uniform cell suspension. Neutral lipids were stained with Nile Red (Thermo Fisher Scientific; excitation/emission 552/636 nm). Briefly, 10 µL of 100 µM Nile Red in dimethyl sulfoxide (DMSO) was mixed with 200 µL of *H. akashiwo* culture supernatant and vortexed for even dispersion; 200 µL of well-mixed cell suspension was then added to yield a final dye concentration of 2.4 µM. Samples were incubated for 15 min in the dark at room temperature. This short incubation and low dye concentration minimized dye exchange or leakage from LDs and avoided measurable perturbation of cell physiology.

For live-cell imaging, no chemical fixation was applied. After staining, a small aliquot was placed on a glass slide, covered with a coverslip, and imaged using an Olympus CKX53 inverted microscope equipped with a FLIR Grasshopper 3 (GS3-U3-41C6C-C) high-resolution color camera. The slide–coverslip geometry provided mild mechanical confinement, allowing cells to remain alive and motile, though typically slowed after approximately 15 min of incubation with dye—facilitating stable imaging. To limit phototoxicity, the excitation LED (552 nm) was kept at a low intensity (relative setting 5). A TRITC-equivalent filter set with a narrow emission band centered near 630 nm and strong rejection beyond ∼ 660 nm was used to minimize chlorophyll autofluorescence background.

Neutral-lipid imaging was performed as paired single-cell measurements. Each cell was first examined in phase-contrast to verify motility and intact morphology (no lysis-like features); immediately thereafter, the same cell was recorded in the fluorescence channel. Cells failing the phase-contrast criteria were excluded from analysis. This paired imaging protocol ensured that the quantified lipid signal originated from viable, morphologically intact cells.

Time-series image stacks were recorded at 16 fps. Across each biological and technical replicate, hundreds of cells were imaged per time point. For statistical comparison and balanced sampling across conditions, a random subsample of 20 cells per biological replicate and per technical replicate was selected for quantitative analysis.

Cell cross-sectional area, LD dimensions (area and volume proxies), and LD spatial distribution were extracted from the image stacks. LD contours were obtained by threshold-based segmentation using custom MATLAB scripts (Image Processing Toolbox) and ImageJ; from each LD, the maximum and minimum Feret diameters were computed. Assuming a prolate-spheroid geometry, Feret diameters were used to estimate an LD volume proxy; the radius of an equivalent-volume sphere was then used to compute a standardized LD cross-sectional area metric for cross-sample comparison. The same segmentation workflow was applied to the cell body to obtain cell cross-sectional area (see Supplementary Figure S3). The normalized lipid size was defined as the ratio of LD area to cell area for each cell. Additional details of the LD quantification pipeline are provided in (Sengupta et al., 2022).

In this study, we focused on single-cell neutral-lipid measurements (Nile Red fluorescence microscopy) alongside population growth and photophysiology. Bulk biochemical assays (e.g., Nile Red fluorimetry, gravimetric lipid extraction, or GC-FAME) were not performed here but are planned for follow-up work to complement the single-cell results.

To quantify the lipid production trends, we define an index of lipid accumulation, $I_{L}$ for each scenario (Figure 4B inset panel) as follows:

$$I_{L}=\frac{{\left[A_{\text{norm}}\right]}_{\text{Perturbed}}}{{\left[A_{\text{norm}}\right]}_{\text{Static}}}$$ (6)

Here ${\left[A_{\text{norm}}\right]}_{\text{Perturbed}}$ and ${\left[A_{\text{norm}}\right]}_{\text{Static}}$ represent the mean normalized lipid area for the perturbed and static populations respectively.

### 2.7 Evaluating changes in photophysiology due to hydrodynamic cues

Key photosynthetic indices, including the maximum quantum yield of Photosystem II (PSII activity or $F_{v}/F_{m}$ ), the maximum relative electron transport rate ( $rETR_{\max}$ ), and non-photochemical quenching ( NPQ ), were measured using a Multi-Color-PAM-II Chlorophyll Fluorometer (Heinz Walz GmbH, Effeltrich, Germany) controlled by *PAMWin* software (v3.22d). Aliquots of 1.5 mL were gently extracted from each culture, transferred into quartz-silica cuvettes (Hellma Analytics, 10 mm path length, 200–2,500 nm spectral range), and dark-acclimated for 5 min prior to measurement. Longer dark-acclimation periods did not alter the results, consistent with previous work (Sengupta et al., 2022). The blue LED channel ( λ=440 nm ) was used for both the measuring light (ML) and the actinic light (AL). Saturation pulses (SP) were applied using the standard MC-PAM protocol to determine the minimal and maximal fluorescence in the dark ( $F_{0}$ , $F_{m}$ ) and, in illuminated samples, the steady-state fluorescence ( $F^{\prime }$ ) and maximal fluorescence under actinic illumination ( $F_{m}^{\prime }$ ). A stepped rapid light-response curve (rapid light curve, RLC) consisting of 16 steps was applied, increasing the actinic irradiance from 0 to 2,500 µmol photons m^-2^ s^-1^. Each step lasted approximately 20 s; for each step, fluorescence was considered to have reached quasi steady-state when the baseline signal $F^{\prime }$ showed no systematic trend over the last few seconds before the saturation pulse, and the corresponding $F_{m}^{\prime }$ values plateaued between successive pulses. The fluorescence parameters were computed using *PAMWin* and verified manually.

The maximum quantum yield of PSII, representing the efficiency of PSII photochemistry under dark-adapted conditions, was calculated as:

$$\frac{F_{v}}{F_{m}}=\frac{F_{m}-F_{0}}{F_{m}}$$ (7)

where $F_{m}$ and $F_{0}$ are the maximum and minimum fluorescence yields in the dark-adapted state, respectively.

The effective quantum yield of PSII under actinic light was calculated as:

$$Y\left(II\right)=\frac{F_{m}^{\prime }-F^{\prime }}{F_{m}^{\prime }}$$ (8)

where $F_{m}^{\prime }$ and $F^{\prime }$ are the maximal and steady-state fluorescence under actinic illumination.

The non-photochemical quenching, representing the fraction of energy dissipated as heat or through non-radiative processes, was computed as:

$$NPQ=\frac{F_{m}-F_{m}^{\prime }}{F_{m}^{\prime }}$$ (9)

The relative electron transport rate (rETR) was determined for each light-curve step as:

rETR=E×Y(II)×0.5 (10)

where E is the actinic irradiance (in µmol photons m^-2^ s^-1^), and the factor of 0.5 accounts for the approximate equal distribution of absorbed light energy between PSI and PSII. Light-response curves of rETR versus E were fitted in *PAMWin* using the built-in EP model (Eilers–Peeters-type photosynthesis function), and $rETR_{\max}$ was taken as the maximum of the fitted curve for each sample.

The NPQ values were obtained directly from the MC-PAM output at each LC step, where *PAMWin* computes NPQ according to Equation 9 using the dark–adapted $F_{m}$ and the step–specific $F_{m}^{\prime }$ values. Thus, for every measurement time point, the LC protocol yielded a full NPQ–PAR series (NPQ as a function of actinic irradiance E ). Because the predefined LC steps did not always include an exact 500 µmol photons m^-2^ s^-1^, the NPQ value at this irradiance ( $NPQ_{500}$ ) was estimated by local least–squares regression of NPQ versus PAR using the two steps bracketing 500 and their nearest neighbours, and the fitted NPQ–PAR curve was evaluated at 500 to obtain $NPQ_{500}$ . Inspection of the NPQ–PAR series for all strains and time points showed smooth, monotonically increasing curves that approached saturation at higher irradiances, consistent with standard rapid light–curve behaviour. The irradiance of 500 µmol photons m^-2^ s^-1^ was selected as a representative actinic light level corresponding to typical near–surface conditions experienced by marine phytoplankton (Sengupta et al., 2022), and $NPQ_{500}$ is used here as a comparative index of non–photochemical dissipation capacity at this ecologically relevant irradiance.

For each time point, the reported values of $F_{v}/F_{m}$ , $rETR_{\max}$ , and $NPQ_{500}$ represent the mean across all biological and technical replicates, together with the corresponding standard deviations. All photophysiological parameters were analyzed over time for both hydrodynamic scenarios. The procedures for MULTI-COLOR-PAM measurements and wavelength-dependent electron-transport analyses follow established protocols (Sengupta et al., 2022; Schreiber et al., 2012; Roháček, 2002), together with the manufacturer’s manual (Heinz Walz GmbH, 2025).

### 2.8 Statistical tests

We conducted paired t-tests to compare the total lipid accumulation, normalized lipid droplet area, and photophysiology parameters for the control populations (no perturbations) as well as the experimental populations (both Scenarios 1 and 2) at regular intervals on the growth curve, spanning early exponential to the stationary growth stages. For the statistical analysis of the growth kinetics (carrying capacities and doubling times) between the control and experimental groups, we utilized two-sample t-tests with the significance level set at P=0.05 . All statistical analyses were performed using GraphPad Prism software.

## 3 Results

### 3.1 Hydrodynamic cues expedite growth but maintain carrying capacity

**FIGURE 2:**
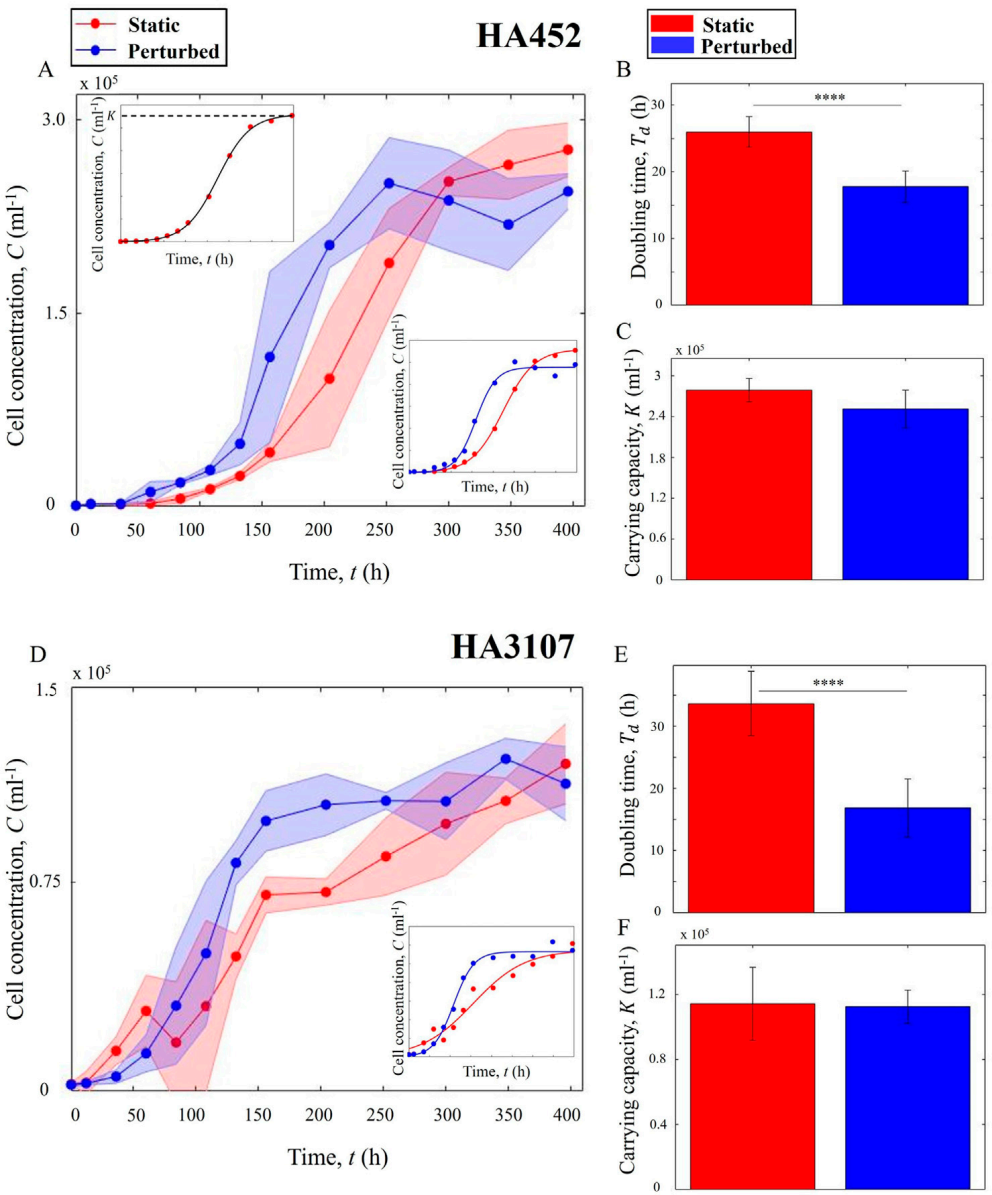
Hydrodynamic cues promote algal growth without impacting population size. **(A)** Comparative growth curves of HA452 under static (red) and hydrodynamic cues (blue). The plot shows the average cell count across all biological and technical replicates. The solid line connects the mean values, while the shaded area indicates the standard deviation. Upper inset shows a fitted logistic curve on sample growth data, where K indicates the carrying capacity. The lower inset presents growth curves fitted by a logistic function for static and perturbed cells. **(B)** Comparative doubling times for the two populations in **(A)**. The bar plots show the mean doubling time for each subset, with error bars representing the standard deviation. The data indicates that hydrodynamic perturbation immediately after inoculation significantly reduces the doubling time ( P< 0.0001), accelerating HA452 cell growth. **(C)** Estimated carrying capacities for the two populations in **(A)** show that the maximum population size remain comparable across both conditions ( P> 0.05). **(D)** Growth curves of HA3107 cultures under static and hydrodynamic cues, the inset plots refer to similar plots as described above. **(E)** Doubling times for the two populations in **(D)** indicate that the hydrodynamic cues significantly reduces the doubling time ( P< 0.0001), while the carrying capacities **(F)** show minimal change ( P> 0.05). For each data point, samples were obtained from three different biological replicates, each sampled at least twice (yielding two technical replicates).

We quantify and compare the growth kinetics of freshly inoculated populations exposed to hydrodynamic cues (Scenario 1) with their control counterparts. Using microscopy and image analysis as detailed in Section 2.4 (Figure 1D), we computed the cell concentration as a function of time. Figures 2A,D present the growth curves for HA452 and HA3107 populations for both the control and hydrodynamically perturbed cases. The shaded region around the solid-line indicates the standard deviation, while the solid line represents the mean value measured across all replicates. We fit a logistic function (bottom right inset panels for each growth curve) to derive their doubling time (Equations 3,4) (Supplementary Figure S1) and the carrying capacity (Equation 3). For HA452, the doubling time drops significantly (Figure 2B), while the carrying capacity remains relatively unchanged (Figure 2C). This indicates that the population exposed to hydrodynamic cues achieve their maximum concentration more rapidly. Mechanical agitation right after inoculation does not inhibit normal biomass yield but significantly decreases the time required to reach maximum biomass, thereby increasing the specific growth rate.

For the HA3107 population (Figures 2E,F), a similar trend was observed: the culture reached carrying capacity markedly earlier than the control. Statistical analysis indicates that the maximum biomass yield did not differ significantly between control and perturbed cells (Figure 2F). However, the fitted specific growth rate r (Equation 3) in perturbed cells was about twofold higher than in controls, and accordingly the doubling time was approximately halved (Figure 2E). These results confirm that initiating hydrodynamic perturbation shortly after fresh inoculation (within a couple of hours) promotes faster growth without altering the final biomass yield.

### 3.2 Onset timing of the hydrodynamic cues tunes the maximum population size

In Scenario 2 of our studies, we investigate the impact of the onset timing of hydrodynamic cues on the growth kinetics and population size. The cell cultures were initially maintained under static conditions, and upon reaching the mid-exponential growth phase ( ≈ 120 h after inoculation), they were exposed to perturbations. This specific time point was selected based on the populations’ physiology, as observed in our current experiments as well as previously reported growth kinetics for HA452 (Sengupta et al., 2017) and HA3107 (Sengupta et al., 2022). Subsequent steps to quantify the growth curve were similar to those outlined in Sections 2 and 3.1.

**FIGURE 3:**
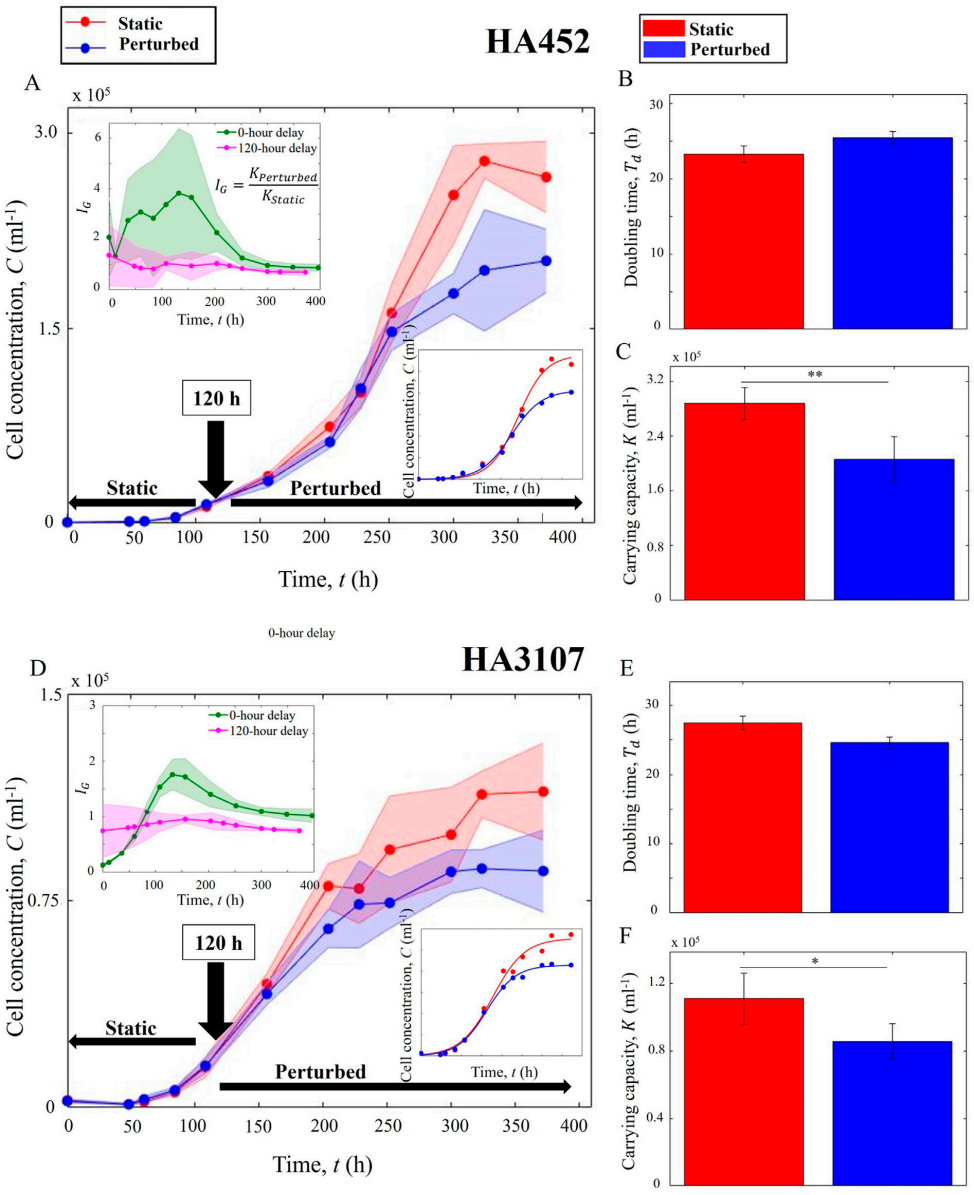
The onset timing of hydrodynamic cues tunes the population size. **(A)** Growth curves of HA452 cultures under Scenario 2 (120-h delay, shown in blue) relative to the control population (red). The transition time point at which the hydrodynamic cues are introduced is shown by the black arrow. Upper inset compares the growth indices for Scenario 1 (0-h delay, green) versus Scenario 2 (120-h delay, pink). The lower inset shows the logistic-fitting on the cell concentration. **(B)** No significant difference between the doubling times of the control and perturbed populations is noted ( P>0.05 ). **(C)** The maximum population size, i.e., the carrying capacity, shown as bar plots (error bars indicate standard deviation), is significantly suppressed in case of the perturbed sample (blue) relative to the control population (red), P<0.01 . **(D)** Growth curves for two subsets of HA3107 cultures, grown under the same conditions as HA452 cells, follow a similar trend. The upper inset illustrates the growth indices, while the lower inset displays the logistic fitting for the cell concentrations. **(E)** Hydrodynamic cues introduced at the mid-exponential growth phase does not impact the doubling time ( P>0.05 ), **(F)** however the carrying capacities for the two sets of populations differ significantly ( P<0.05 ). For each data point, samples were obtained from three different biological replicates, with each sample having two technical replicates.

As shown in Figure 3, the carrying capacity varied significantly between the control and the perturbed populations for both strains (panels C and F of Figure 3). On the other hand, the doubling times remained unaffected, as depicted in Figures 3B,E respectively for HA452 and HA3107. This is in contrast to the Scenario 1 (cells were subjected to perturbation immediately after inoculation, i.e., 0-h delay), where the carrying capacity remained stable while the doubling time dropped significantly (Figure 2). Conversely, in the Scenario 2, a significant reduction in carrying capacity is noted, with non significant variation in the doubling times.

During the initial phase, the negative slope for the 0-h delay plot is steeper than for the 120-h delay plot, indicating that cells under immediate perturbation have limited ability to grow, produce biomass and acclimate. Consequently, these cells produced less new biomass compared to the control cells. After approximately 1 day, the slope of the plot remained positive up to around 150 h. This indicates that cells subjected to hydrodynamic perturbation immediately after inoculation grew favourably and produced more biomass until reaching their mid-exponential phase. Due to their rapid growth, even when the slope of their growth index became negative again after 150 h, their cell concentration remained higher than that of the static cells until around 300 h. The growth index for the 0-h delay perturbation remained higher than 1 throughout the stationary phase, decreasing to approximately 0.85 after 400 h (late stationary phase). In case of Scenario 2 (120-h delay), $I_{G}$ fluctuated around 1 before the transition point. When the cells were subjected to hydrodynamic perturbation, $I_{G}$ remained marginally above 1 for around 100 h, potentially due to homogenized nutrient distribution, which facilitated higher nutrient access for the cells (Fuentes-Grünewald et al., 2013). Subsequently, the index reduced to less than 1, eventually decreasing to approximately 0.69.

Taken together, these results indicate that algal populations, when exposed to hydrodynamic cues early on in their growth phase, proliferated at a faster rate, achieving the same carrying capacity within a shorter time. In contrast, delayed exposure to hydrodynamic cues allowed the population to grow at comparable rates during the exponential phase but transitioned to their stationary phase sooner than the control samples. Thus, freshly inoculated motile cells grown under hydrodynamic perturbations increased their productivity relative to the control cells grown under static conditions. Comparing $I_{G}$ values for HA452 and HA3107 for the 0-h delay scenario, a longer adaptation period is noted for HA3107 when exposed to hydrodynamic perturbations. From around 96 h–150 h (the exponential growth phase), the perturbed cells exhibited higher biomass production while continuously subjected to hydrodynamic perturbation. After 150 h, the slope turned negative, indicating a higher growth rate for the control cells compared to the perturbed ones. Ultimately, this resulted in equivalent carrying capacities for both subsets of cells in the 0-h delay scenario.

For the Scenario 2, the growth index exhibited an immediate increase following the introduction of the perturbation, reaching its maximum value. This increase is likely attributable to a more uniform nutrient distribution within the cultures of the perturbed populations. Previous studies have demonstrated that cellular stress may cause HA3107 to exhibit diffusive than ballistic behavior (Sengupta et al., 2022), which can further restrict their access to nutrients. The growth was subsequently suppressed due to hydromechanical forces, stabilizing at around 0.74. This stabilized value is higher than the corresponding index for HA452 cells, indicating that HA3107 cells are less adversely affected by the 120-h delay perturbation scenario. The onset timing of hydrodynamic cues thus offers a novel handle to regulate algal growth kinetics. While the extent of these responses are strain-dependent, the observed trends were found to be similar for both the growth rate as well as the carrying capacity.

### 3.3 Algal lipid production depends on the onset timing of hydrodynamic cues

**FIGURE 4:**
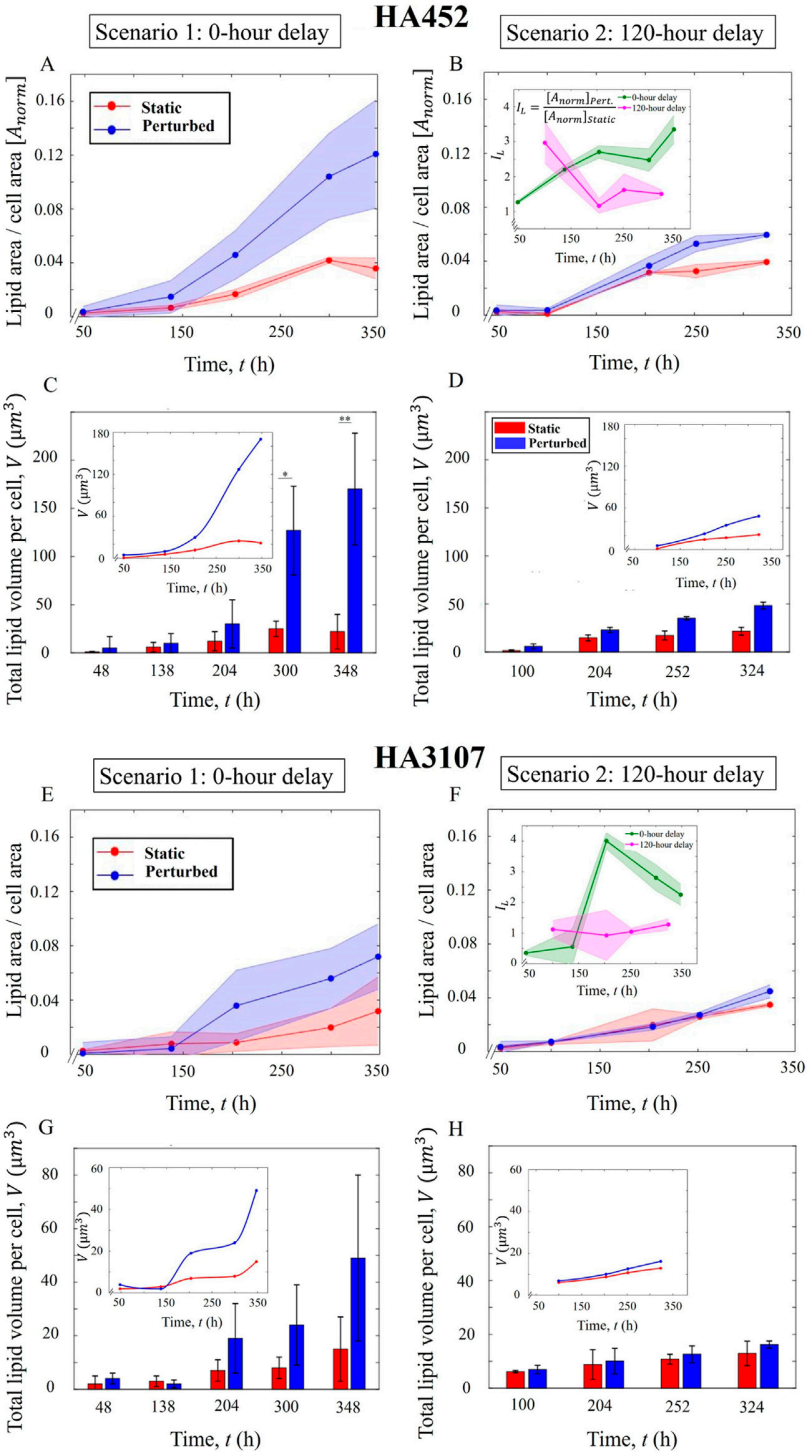
Onset timing of hydrodynamic cues modulates enhancement of lipid production. **(A)** Normalized lipid area across the growth phases under Scenario 1 (0-h delay), for HA452. Solid curve represents the average ratio over the replicates, while the shaded area shows the standard deviation. Significant increment of lipid production ( >3 -fold, P<0.001 ) is noted. **(B)** Delayed exposure to hydrodynamic cues (120-h delay) results in a less pronounced increment of lipid ( P<0.05 ). The inset shows the index of lipid accumulation, $I_{L}=\frac{{\left[A_{\text{norm}}\right]}_{\text{Perturbed}}}{{\left[A_{\text{norm}}\right]}_{\text{Static}}}$ for both scenarios across the growth phase. **(C)** Total lipid volume per cell shows a statistically significant increase compared to the control cells ( P<0.01 ) during the stationary phase. The inset captures the trend during the course of experiment. **(D)** Total lipid volume per cell for the 120-h delay scenario exhibited lower increment relative to the 0-h delay case. The inset shows the corresponding trends during the course of the experiment. **(E)** Normalized lipid area for HA3107 under Scenario 1 (0-h delay) shows a marginal increment ( p=0.555 ), while for Scenario 2 **(F)**, an increase in the normalized lipid area was noted, which was lower compared to the 0-h delay scenario. The inset shows the variation of $I_{L}$ which was around 2.25 at the end, indicating larger lipid droplets for the perturbed cells. For Scenario 2, $I_{L}$ remained ≈ 1 throughout, with a slight increase during the stationary growth stage for both control and perturbed cells. **(G)** Total lipid volume per cell for HA3107 cells under 0-h delay scenario showed no significant enhancement ( p=0.0745 ). The inset shows the trend over the entire duration of the experiment. **(H)** The total lipid volume per cell in HA3107 under the 120-h delay condition showed marginal enhancement.

Lipid accumulation was systematically monitored throughout both experimental scenarios (0-h delay and 120-h delay scenarios). Cell samples were collected at regular intervals from all culture tubes, in accordance with the protocols outlined in Section 2.6. Unlike previous studies (Fuentes-Grünewald et al., 2013; Lou et al., 2020; Yang et al., 2023), we have quantified lipid accumulation at the single cell level using epifluorescent microscopy (Figure 1C), with additional validation using bright-field imaging at ×100 magnification (Figure 1D, inset). For the 0-h delay scenario (Figure 4A), the normalized lipid area was consistently higher for the populations exposed to the hydrodynamic cues, indicating a sustained enhancement of the lipid production across all time points (while the cell area remained constant, Supplementary Figure S3A). Despite similar biomass yields between the control and perturbed cells, as shown in Figure 2C, the normalized lipid area in perturbed cells increased notably during the stationary phase, resulting in an almost 4-fold enhancement upon exposure to hydrodynamic cues ( P<0.01 ) after 350 h. While nutrient limitation affects both the control and perturbed cell cultures by the time they reach the early stationary phases (Sengupta et al., 2022), nutrient stress alone cannot account for the significantly higher lipid accumulation observed in the populations exposed to hydrodynamic cues. Depletion of nitrates and phosphates (see Supplementary Figure S5) are known to induces cellular stress, leading to increased lipid production and accumulation (Sengupta et al., 2022), our results indicate that hydrodynamic cues allow significantly higher enhancement beyond the nutrient-limited lipogenesis.

For the Scenario 2 (120-h delay, Figure 4B), the normalized lipid area remained comparable between control and perturbed cells for approximately 100 h following the onset time point. However, upon reaching the stationary growth phase, a significant increase in the normalized lipid area was observed for the perturbed cells compared to the control cells ( P<0.05 ). This increase in lipid accumulation was less pronounced than in Scenario 1: an increase of 150% compared to an increase of ≈ 400% observed in the 0-h delay scenario.

In the 0-h delay case, $I_{L}$ showed a steady increment, with the steeper slope observed during the exponential phase. As demonstrated in Section 3.1, HA452 cells in their exponential phase under hydrodynamic perturbation displayed accelerated growth and a significantly reduced doubling time compared to static cells. The lipid index indicates that these cells while growing at a faster rate than static cells, were concurrently accumulating more neutral lipids. The formation and storage of lipid droplets are biomarkers of cellular stress (Sengupta et al., 2022), which is known to suppress growth (Lou et al., 2020). However, our results suggest an acclimation strategy whereby the HA452 population subjected to a stressful environment from the outset, allowing them to thrive and accumulate lipids while sustaining normal growth.

As the population entered the early stationary phase, the $I_{L}$ stabilised due to a drop in the cell concentration of the perturbed population relative to the static population. It then sharply increased starting from the mid-stationary phase, where a combination of hydrodynamic and nutrient-limitation induced cellular stress in both populations (Sengupta et al., 2022). For the 120-h delay scenario, the lipid accumulation index exceeded 1 prior to the hydrodynamic cues, with both populations initially exposed to identical environmental conditions. Despite introduction of hydrodynamic cues 120 h post-inoculation, $I_{L}$ continued to drop for 100 h post-perturbation, likely a more homogeneous nutrient distribution (due to shaking), thereby offering temporary adjustment to the hydrodynamic perturbation. $I_{L}$ eventually stabilized at around 1 after 100 h post-perturbation, as also reflected in the trends observed for the growth index (Figure 3A, inset) and the normalized lipid area (Figure 3B), suggesting that the cells were able to maintain their physiological state for ≈ 100 h following exposure to the hydrodynamic stressor. Overall, the results indicate that algal populations exposed to hydrodynamic cues freshly after inoculation, are not only able to successfully maintain their normal growth, they also yield higher lipid volumes (Figures 4A, C). Conversely, hydrodynamic cues applied on populations grown under static conditions for some time (here, until mid-exponential phase) led to reduced biomass alongside significantly lower lipid accumulation (Figures 4B, D).

**FIGURE 5:**
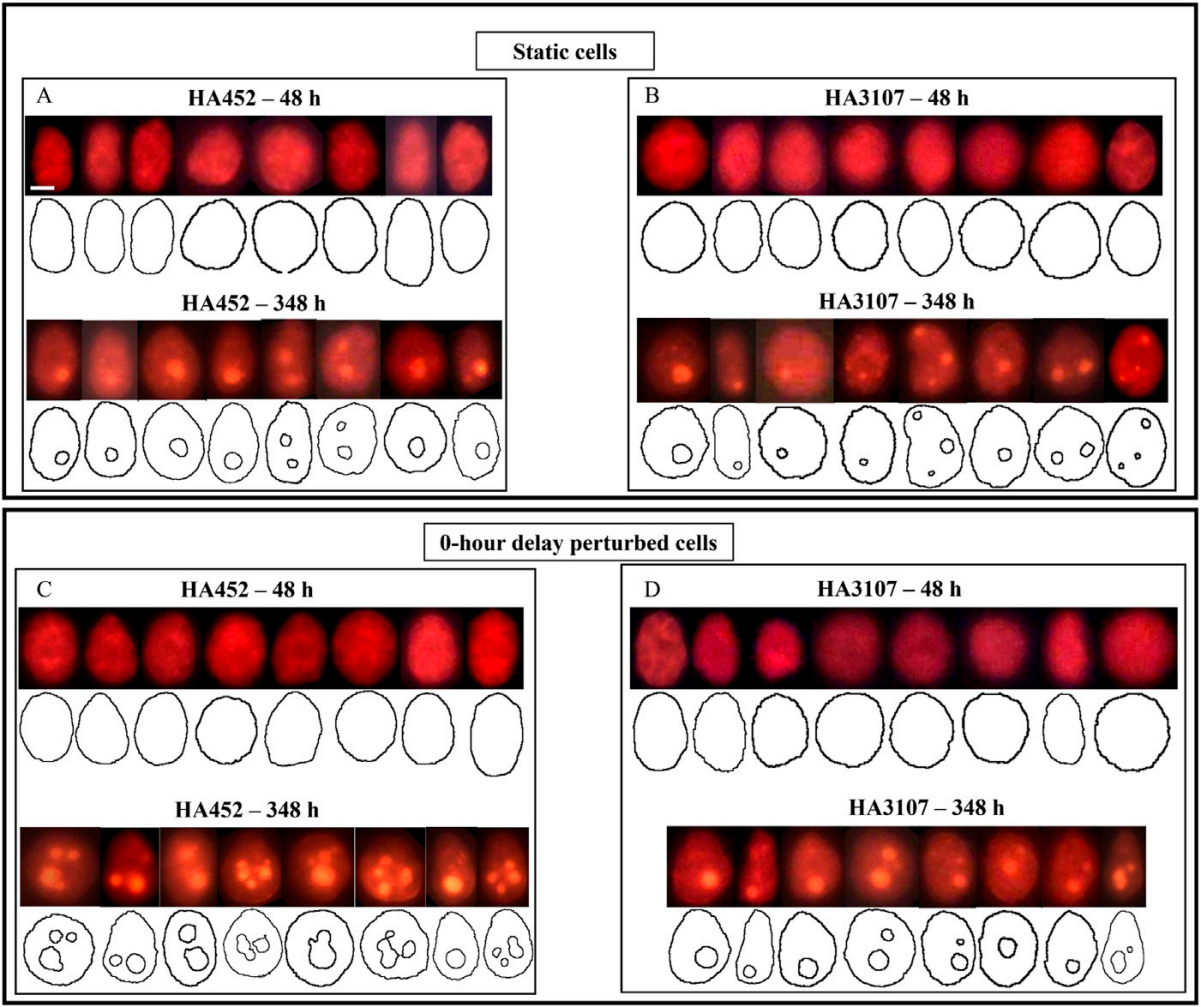
Scenario 1 (0-h delay): Quantifying lipid production in *Heterosigma akashiwo* using single cell fluorescence microscopy. **(A**–**D)** Representative fluorescent images, which were analysed to obtain the lipid droplet (LD) dimensions and cell contours for both strains of *H. akashiwo*, shown here for the Scenario 1 (0-h delay), for early exponential (48 h) and stationary growth phases (348 h). The neutral LDs appear bright orange. Control (static) populations are shown in **(A,B)** for HA452 and HA3107 respectively, while corresponding hydrodynamically perturbed populations are shown in **(C,D)**. Under static conditions, the average lipid accumulation increased from $1.0\pm 0.5\mu {\text{m}}^{3}/\text{cell}$ at 48 h to $22.0\pm 18.1\mu {\text{m}}^{3}/\text{cell}$ at 348 h ( n=60 cells) for HA452; and from $2.0\pm 3.0\mu {\text{m}}^{3}/\text{cell}$ at 48 h to $15.2\pm 12.0\mu {\text{m}}^{3}/\text{cell}$ at 348 h ( n=60 cells) for HA3107. **(C)** HA452 population subjected to 0-h delay perturbation at 48 h (top row) and 348 h (bottom row), reveal an increase in cytoplasmic lipid accumulation from $5.0\pm 12.0\mu {\text{m}}^{3}/\text{cell}$ at 48 h to $170.3\pm 58.6\mu {\text{m}}^{3}/\text{cell}$ at 348 h ( n=60 cells). **(D)** HA3107 under 0-h delay perturbation showed an increase in cytoplasmic lipid accumulation from $4.2\pm 2.0\mu {\text{m}}^{3}/\text{cell}$ at 48 h to $49.1\pm 31.5\mu {\text{m}}^{3}/\text{cell}$ at 348 h ( n=60 cells). The scale bar represents 5 μ m.

**FIGURE 6:**
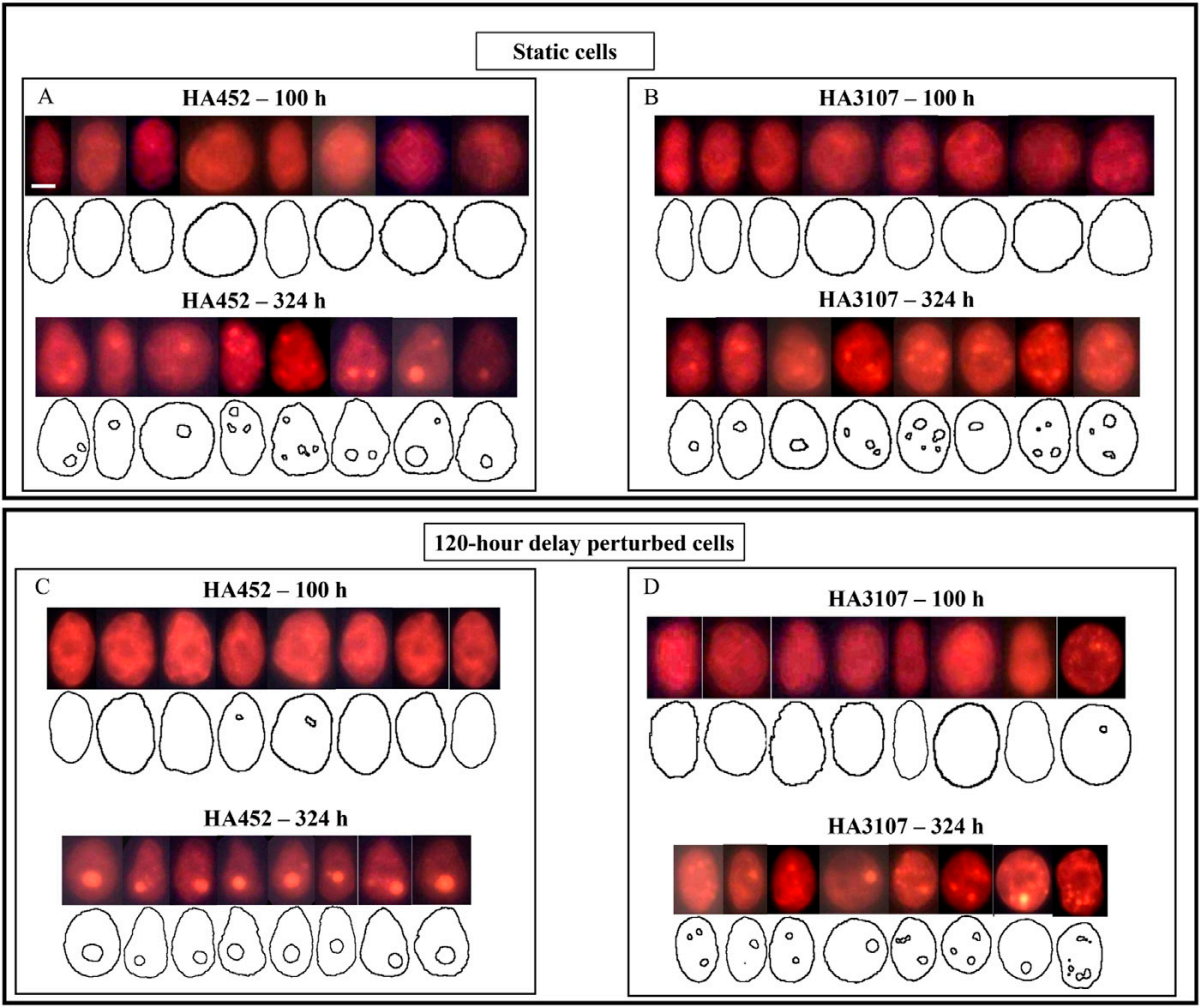
Scenario 2 (120-h delay): Quantifying lipid production in H. akashiwo using single cell fluorescence microscopy. **(A**–**D)**: Representative fluorescent images, which were analysed to obtain the lipid droplet dimensions and cell contours for both strains of *H. akashiwo*, shown here for the Scenario 2 (120-h delay), for exponential 100 h and stationary growth phases (324 h). The neutral LDs appear bright orange. Control (static) populations are shown in **(A,B)** for HA452 and HA3107 respectively, while corresponding hydrodynamically perturbed populations are shown in **(C,D)**. The average lipid accumulation increased from $1.4\pm 0.8\mu {\text{m}}^{3}/\text{cell}$ at 100 h to $21.6\pm 4.1\mu {\text{m}}^{3}/\text{cell}$ at 324 h ( n=60 cells) for static HA452 populations. Correspondingly, lipid accumulation varied from $6.2\pm 0.4\mu {\text{m}}^{3}/\text{cell}$ at 100 h to $12.9\pm 4.5\mu {\text{m}}^{3}/\text{cell}$ at 324 h ( n=60 cells) for HA3107 under static condition. **(C)** Upon exposure to hydrodynamic cues after a 120-h delay, HA452 cells had accumulated $5.8\pm 2.6\mu {\text{m}}^{3}/\text{cell}$ at 100 h, which significantly increased ( P<0.05 ) to $48.2\pm 3.5\mu {\text{m}}^{3}/\text{cell}$ at 324 h ( n=60 cells). **(D)** Lipid accumulation in HA3107 cells subjected to a 120-h delay perturbation increased marginally from $6.9\pm 1.6\mu {\text{m}}^{3}/\text{cell}$ at 100 h to $16.2\pm 1.3\mu {\text{m}}^{3}/\text{cell}$ at 324 h ( n=60 cells). The scale bar represents 5 μ m.

For HA3107 cells, the normalized lipid area for both control and perturbed cells is depicted in Figure 4E for the 0-h delay scenario and in Figure 4F for the 120-h delay scenario. For the Scenario 1, lipid accumulation increased more than that for the control populations, at around 140 h after inoculation. From this time point onward, the accumulated lipid in the perturbed cells remained consistently higher than in the control cells. Although this parameter (Figures 4E, G) more than doubled in the perturbed cells compared to the control ones, we could not establish statistically significant difference. Therefore, for HA3107 cells under the 0-h delay scenario, the cells grew at a higher rate with a notably reduced doubling time, resulting in a similar biomass over a shorter timescale, with more than double the amount of accumulated lipid. For Scenario 2 (Figure 4F, H), increase in the lipid accumulation did not reach the corresponding value observed for the 0-h delay scenario. Taken together, reduction of the delay time (i.e., an early onset time for hydrodynamic cues) produced more cells with a higher amount of algal lipids, providing a tractable parameter space to enhance algal lipid accumulation. Furthermore, the role of hydrodynamic cues on the onset and volume of lipid accumulation is strain-dependent. As presented in Figures 5 and 6, a markedly higher accumulation of lipid droplets is observed for HA452 relative to HA3107, particularly in populations which were exposed to hydrodynamic cues right after inoculation. The strain-specific difference in the accumulated lipids was less pronounced when the onset time for hydrodynamic perturbations was delayed.

### 3.4 Onset timing of hydrodynamic perturbation impacts algal photo-physiology

Hydrodynamic cues impact photo-physiology (Carrara et al., 2021), yet the role of the onset timing on photo-physiology and possible induction of cellular stress remains unexplored. We quantify the maximum quantum yield of Photosystem II (PSII) photochemistry, represented by the ratio of maximum variable fluorescence $\left(F_{v}\right)$ to maximum fluorescence $\left(F_{m}\right)$ of chlorophyll $\left(F_{v}/F_{m}\right)$ (Sengupta et al., 2022). This ratio (Equation 7), or maximum quantum yield, has been extensively utilized in numerous studies as a standard metric for photosynthetic efficiency and indicator of cellular fitness (de la Rosa et al., 2023). Using a Multi-Color Pulse Amplitude Modulation (PAM) Chlorophyll Fluorometer (see Materials and Methods, Section 2.7), we carry out a time series measurement of photo-physiology for the control and perturbed populations under both hydrodynamic scenarios described previously.

**FIGURE 7:**
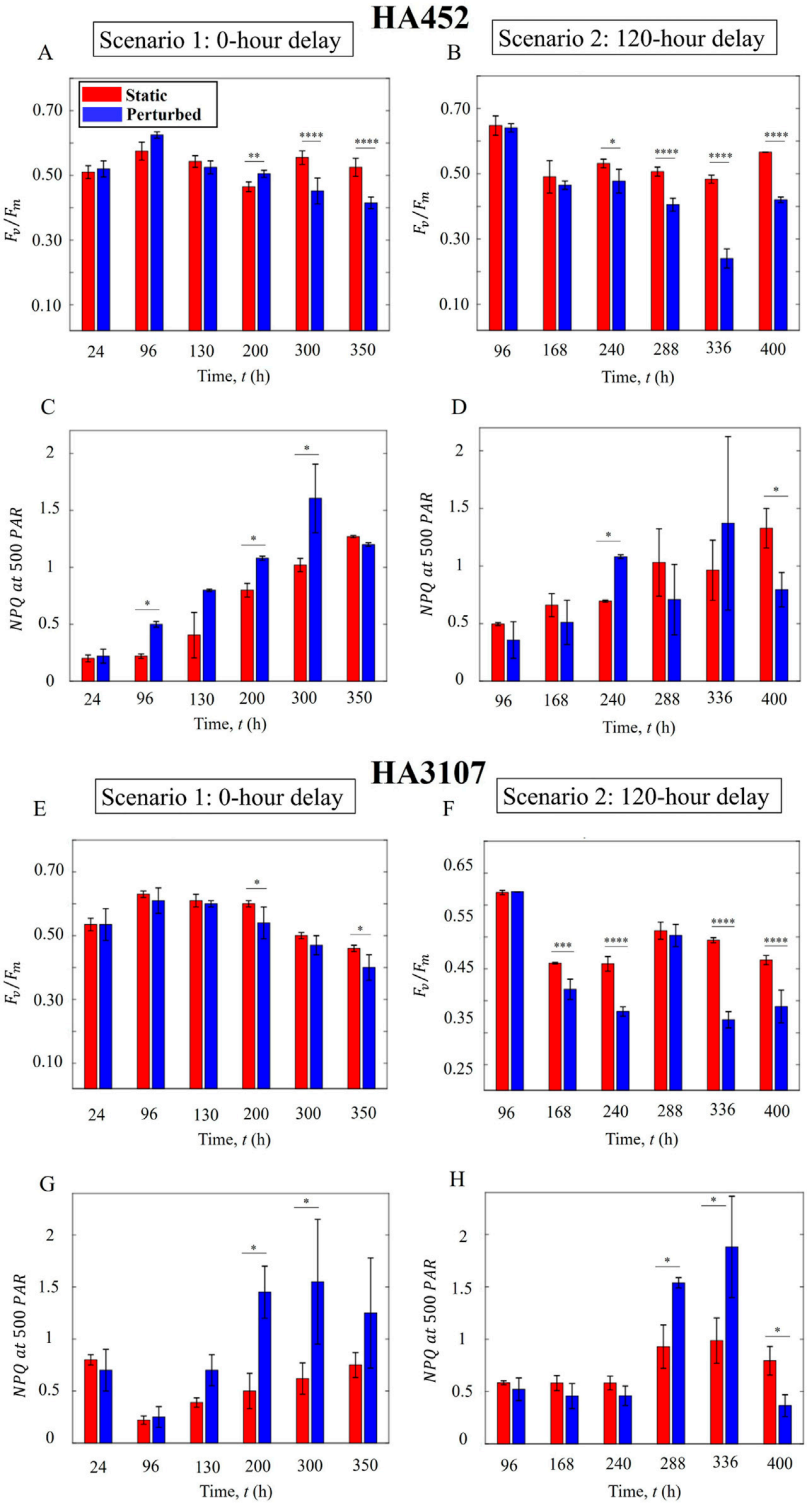
Onset timing of hydrodynamic perturbation impacts algal physiology. **(A)** Photosynthetic performance of HA452 under Scenarion 1: Perturbed cells exhibited comparable photosynthetic activity relative to static cells for up to 11 days, thereafter dropping significantly ( P<0.0001 ) around 300 h post-inoculation. **(B)** Scenario 2: Following the initiation of hydrodynamic cues, a significant decrease in photosynthesis was observed within 5 days ( P<0.0001 ). **(C)** HA452 NPQ values show comparable values initially, which later increased siginificantly ( P<0.05 ), eventually reaching a plateau before decreasing. **(D)** NPQ values of HA452 cells under the 120-h delay scenario. **(E)** Photosynthetic efficiency of HA3107 cells under the 0-h delay experimental condition. The perturbed cells maintained comparable photosynthetic efficiencies to the control cells throughout the experiment until a significant reduction was noted during stationary phase. ( P<0.05 ). **(F)** Photosynthetic efficiency of HA3107 cells under the 120-h delay experimental condition: photosynthetic efficiency was significantly affected, with a marked decline observed at 168 h ( P<0.001 ). **(G)** HA3107 NPQ values under the 0-h delay scenario, and under the 120-h delay scenario **(H)**. The NPQ value never increased in the perturbed cells, while $F_{v}/F_{m}$ and $ETR_{\text{max}}$ were significantly reduced, indicating their inability to self-regulate photophysiological responses to the hydrodynamic perturbations.

Figures 7A,B present the photosynthetic efficiency of HA452 under the 0-h and 120-h delay scenarios, respectively. Populations exposed to hydrodynamic cues immediately post-inoculation (0-h delay) yield comparable photosynthetic efficiencies up to 200 h (Figure 7A), and thereafter showed significant decline in performance spanning the stationary growth phase. On the other hand, the corresponding $ETR_{\text{max}}$ (Equations 8, 10) (see Supplementary Figure S4) remained within the range observed for the control populations. This constancy suggests that, for at least the initial 200 h, the cells neither compensated for the hydrodnamic perturbation nor responded to increased energy demands to maintain their photosynthetic performance (Stewart et al., 2015). Consequently, their photo-physiological status is considered stable. However, beyond 200 h, both the maximum quantum yield of Photosystem II and $ETR_{\text{max}}$ exhibited declines, indicating cellular stress or damage to the photosynthetic apparatus.

The non-photochemical chlorophyll fluorescence quenching (NPQ) (Equation 9), an indicator of the excess absorbed light energy that is ultimately dissipated after conversion into heat (Ji et al., 2024), provides insights into the protective mechanisms against light-induced stress (Hennige et al., 2013). By interpolating the NPQ at 500 $\mu \text{mol}/\left({\text{m}}^{2}/\text{s}\right)$ akin to the light intensity typically observed at the ocean surface (Sengupta et al., 2022), we evaluate the NPQ values across all samples utilizing the series of NPQ values as a function of PAR recorded by the PAM fluorometer (Materials and Methods, Section 2.7). As depicted in Figure 7C, freshly inoculated HA452 exposed to hydrodynamic cues (0-h delay) showed a consistently higher NPQ values over the growth phases. Notably, after around 200 h, there was a substantial increase in NPQ values for the perturbed cells, which plateaued around 300 h. Thereafter NPQ values declined, while for the control case, the NPQ values continued to rise. The NPQ variations align with the $F_{v}/F_{m}$ and $ETR_{\text{max}}$ trends, both of which also dropped after ≈ 300 h. This suggests that around 300 h post-inoculation, the photosynthetic apparatus experience damage, while prior to this, the photosynthetic machinery self-regulated to mitigate stress, maintaining the overall efficiency of the photosynthetic process. This adaptive mechanism enables algal species to balance light utilization for photosynthesis with protection against stress-induced damage, thus ensuring survival and performance under challenging environmental conditions.

In the case of HA3107 cells, we observed a similar trend, but more pronounced. While the $F_{v}/F_{m}$ were comparable initially (Figure 7E), a significant drop was noted around 200 h post-inoculation. Throughout this, the $ETR_{\text{max}}$ remained stable (see Supplementary Figure S4), suggesting that the energy demand and overall photosynthetic efficiency of the perturbed cells were maintained similarly to the control cells, despite the intermittent drop in $F_{v}/F_{m}$ . These observations are corroborated also by the NPQ values, which were comparable initially (Figure 7G) but increased significantly at around 200 h. Subsequently, NPQ values declined, likely due to damage to the photosynthetic machinery, supported further by the trends in population growth and intracellular lipid accumulation. Overall, the variations in these three parameters between the perturbed and control cells suggest that the perturbed cells under the 0-h delay scenario exhibited a self-regulated photo-physiology for at least the initial 300 h post-inoculation.

Delayed introduction of the hydrodynamic cues (120-h delay scenario) impacted the photo-physiology adversely, as indicated by the lower $F_{v}/F_{m}$ values (Figure 7B). A significant decline was observed at 240 h post-inoculation, i.e., around 120 h after the onset of hydrodynamic perturbation. The timing of this significant reduction in photosynthetic efficiency (compared to the 0-h delay setting) suggests that the photosynthetic apparatus takes longer to get impaired if cell populations are exposed to the hydrodynamic cues early on in their growth phase. Furthermore, the variations in $ETR_{\text{max}}$ of HA452 cells under the 120-h delay setting (see Supplementary Figure S4) corresponded closely to the $F_{v}/F_{m}$ variations. While the $ETR_{\text{max}}$ values remain comparable to those of the control population prior to the application of hydrodynamic perturbation, both $F_{v}/F_{m}$ and $ETR_{\text{max}}$ decreased thereafter, indicating that the cells were experiencing a relatively stressful condition, resulting in a reduced efficiency of the photosynthetic apparatus.

The NPQ values for HA452 under the 120-h delay scenario (Figure 7D) remained comparable between the control and perturbed cells for at least the initial 240 h. Following the application of hydrodynamic perturbation, the NPQ values ranged within those of the control populations, in agreement with the growth and lipid production trends. Shortly after, the NPQ values for the perturbed populations increased, contrasting with the decreases observed in $F_{v}/F_{m}$ and $ETR_{\text{max}}$ . Specifically, at 240 h post-inoculation (i.e., 120 h after the onset of perturbation), the energy demand decreased as indicated by the descending trends in $ETR_{\text{max}}$ and $F_{v}/F_{m}$ , the NPQ values increased as a protective response. Subsequently, all three parameters ( NPQ , $F_{v}/F_{m}$ , and $ETR_{\text{max}}$ ) decreased, indicating damage to the photosynthetic apparatus. Finally, for HA3107, delayed introduction of the hydrodynamic cues resulted in an immediate and pronounced reduction in photosynthetic efficiency following the introduction of the perturbation (Figure 7F). A similar pattern was observed in their $ETR_{\text{max}}$ (see Supplementary Figure S4), showing a decline approximately 150 h after perturbation. This indicates that photosynthetic efficiency and energy demand were suppressed relatively quickly after exposure to hydrodynamic perturbation. Concurrently, NPQ values (Figure 7H) significantly increased ( P<0.05 ) in the perturbed cells, reflecting enhanced protection against the stressful conditions. However, despite this response, it was insufficient to sustain efficient photosynthetic performance.

## 4 Discussions

Hydrodynamic forcing at an energy dissipation rate of $9.53\times 10^{-4}\;Wkg^{-1}$ —about an order of magnitude above strong wind-driven mixed layers (Sengupta et al., 2017)—produced marked, timing-dependent physiological responses in *Heterosigma akashiwo*. In the immediate-onset (0-h delay) treatment, strain HA452 entered exponential growth more rapidly and sustained a growth index >1 for nearly 150 h before declining toward stationary phase. Even after mid-exponential slowdown, HA452 biomass remained higher than the static control until ∼300 h, ending at a final growth index of 0.85 in stationary growth phase. In contrast, the same hydrodynamic load applied at mid-exponential phase (120-h delay) curtailed biomass accumulation, with growth index falling below 1 and ending at 0.69.

These growth trajectories translated into distinct lipid outcomes. In HA452, continuous shaking from inoculation produced a nearly fourfold ( ∼ 3.7 × ) ncrease in neutral-lipid volume compared to control static cells by 350 h, despite final biomass being comparable to static populations. When the perturbation was delayed to 120 h, lipid accumulation was only ∼ 1.5 × . This genotype-specific effect was confirmed by HA3107, where control and perturbed cultures showed overlapping growth curves, negligible changes in doubling time, and lipid increases rarely reaching statistical significance—though early perturbation still more than doubled lipid content ( ∼2.3 -fold) relative to control.

Furthermore, our volumetric analyses revealed that HA452 cells under the 0-h delay regime accumulated on average ∼ 180 μm^3^ of neutral lipids per cell within 350 h. This corresponded to an average lipid production rate of at least 0.5 μm^3^ h^−1^ per cell, with peak rates reaching 0.7 μm^3^ h^−1^ in some replicates—well above the population mean. For comparison, under static conditions in our previous work (Sengupta et al., 2022), the maximum rate of lipid production was 0.04 μm^3^ h^−1^ per cell, an order of magnitude lower. Likewise, the maximum lipid droplet volume ( $V_{\text{LD}}$ ) reached only 15 μm^3^ per cell in the static regime, more than tenfold smaller than the 180 μm^3^ observed here. These quantitative gains highlight the substantial potential of early-onset hydrodynamic forcing for enhancing neutral-lipid yields in motile raphidophytes.

The photophysiological patterns are consistent with—but do not by themselves prove—a mechanistic basis for why early hydrodynamic perturbation can preserve fitness while enhancing lipid yield. In HA452, elevated NPQ soon after inoculation indicates active dissipation of excess excitation, while $F_{v}/F_{m}$ and ${ETR}_{\max}$ remained comparable to controls for ∼ 200–250 h; subsequent declines across all three parameters suggest exhaustion of protective capacity. Although we did not directly measure ${\text{Ca}}^{2+}$ signaling, ROS, or redox fluxes, a mechanism consistent with prior studies is that shear-induced membrane deformation triggers ${\text{Ca}}^{2+}$ influx via mechanosensitive channels (Cho and Shin, 2016; Min et al., 2014), activates NADPH oxidase, and elicits a moderate ROS pulse (Yuan et al., 2024). Such signals can reroute carbon toward fatty-acid synthesis and thylakoid-lipid recycling (Wang et al., 2023), providing an ATP/NADPH sink that enables regulated triacylglycerol storage rather than passive lipid accumulation secondary to damage (Saroussi et al., 2017).

The dependence on onset timing is consistent with the metabolic state at the moment of perturbation. When mixing began immediately after inoculation (0-h delay), nutrients were still replete and populations sustained growth while accumulating neutral lipids; photophysiological metrics (elevated NPQ with preserved $F_{v}/F_{m}$ and ${ETR}_{\max}$ for ∼ 200–250 h) indicate that cells accommodated the forcing without early photodamage. By contrast, initiating mixing at mid–exponential phase (120-h delay)—after resources had been largely committed to division—coincided with faster declines in $F_{v}/F_{m}$ and ${ETR}_{\max}$ and with biomass penalties within ∼ 120 h. Consistent with this shift in physiological state, neutral-lipid gains under late onset were modest ( ∼ 1.5 × ) relative to early onset ( ∼ 3.7 × ).

In complementary experiments spanning additional onset times (to be reported elsewhere), delaying hydrodynamic forcing to later growth stages consistently reduced the algal fitness. Additionally, no significant enhancement of lipid accumulation could be observed. In other words, the late onset of the hydrodynamic forcing imposed a larger fitness cost, while failing to enhance lipid accumulation, indicating that the onset timing is a primary determinant of the trade-off.

Ecologically, the laboratory shear mimics episodic coastal turbulence during storms. Motile raphidophytes that convert turbulence into rapid neutral lipid accumulation may better endure fluctuating light and nutrient regimes, potentially outcompeting other species. Strain-level variation, as seen between HA452 and HA3107, highlights biodiversity’s role in buffering phytoplankton communities against physical disturbances under climate change.

This study identifies a scalable intervention—precisely timed, low-intensity mechanical mixing—that partially decouples the canonical trade-off between biomass accumulation and neutral-lipid storage. Although the present work is constrained by bench-scale vessels, a focus on neutral lipids, two representative strains, and a 2-week observation window, systematic extension to transcriptomic and lipidomic profiling, pilot-scale validation, and expanded strain panels would enable mechanistic resolution and assess industrial translatability, thereby positioning hydrodynamic timing as a practical lever in sustainable algal biofuel production.

## 5 Conclusion

Our study demonstrates that hydrodynamic perturbation, if timed appropriately, could play an important role in enhancing algal lipid production in motile species, while maintaining biomass generation and photo-physiology, thus overall fitness. Immediate exposure to hydrodynamic perturbation after inoculation in fresh nutrient media enhances lipid production, while a delayed exposure leads to reduced growth and suboptimal lipid storage over the long run. While biofuel production has primarily relied on non-motile species, hydrodynamic manipulation of motile microalgae can offer a distinct advantage. Previous studies have indicated deleterious effects of hydrodynamics on motile species, potentially leading to flagellar and body wall damage, reduced lipid production, and impaired organelle functioning (Thomas and Gibson, 1990). Yet, fluid flow–a prevalent physical factor in the ecology of phytoplankton–is critical for microalgal growth and fitness as conceptualised in Margalef’s ‘mandala’ (Margalef, 1978; Sengupta et al., 2017). Following the mandala, hydrodynamic cues, as shown in the current study, can be leveraged as biophysical stressors for motile species and combined strategically with nutrient or light limitations to optimise algal biofuel yields under sustained biomass production (Chowdury et al., 2020; Ding et al., 2021; García Camacho et al., 2000).

Few studies have explored the hydrodynamic impacts simultaneously on algal growth, photo-physiology and lipid production, in particular for *Heterosigma akashiwo*. Our work does so in a strain-specific manner, demonstrating a 370% and 230% increases in lipid accumulation for HA452 and HA3107 strains, respectively, under appropriately timed hydrodynamic perturbations. Although reactive oxygen species (ROS) measurements were not performed, indirect indicators, such as increased NPQ and suppressed photosynthetic activity, suggest that physiological stress triggered by hydrodynamics mediated lipid production. The findings presented here establish hydrodynamic perturbation as a promising yet underutilized strategy for enhancing lipid accumulation in motile microalgae, particularly for industrial biofuel applications. Future research should explore how other motile species, typically used in industrial biofuel production, respond to hydrodynamic cues and their onset timing. Furthermore, the scalability of the hydrodynamic approach and its integration with lipid extraction methodologies may be investigated to maximize biofuel extraction in an energy-efficient, sustainable manner.

## Data Availability

The raw data supporting the conclusions of this article will be made available by the authors, without undue reservation.
