# Supplementary material for: Tuning lipid accumulation and fitness of motile algae via hydrodynamic cues
Source: Front Bioeng Biotechnol. 2026 Jan 5;13:1722499. doi: 10.3389/fbioe.2025.1722499 (PMC12812883; doi:10.3389/fbioe.2025.1722499)
Supplement: Supplementary file 1 [file DataSheet1.pdf]

Supplementary Material

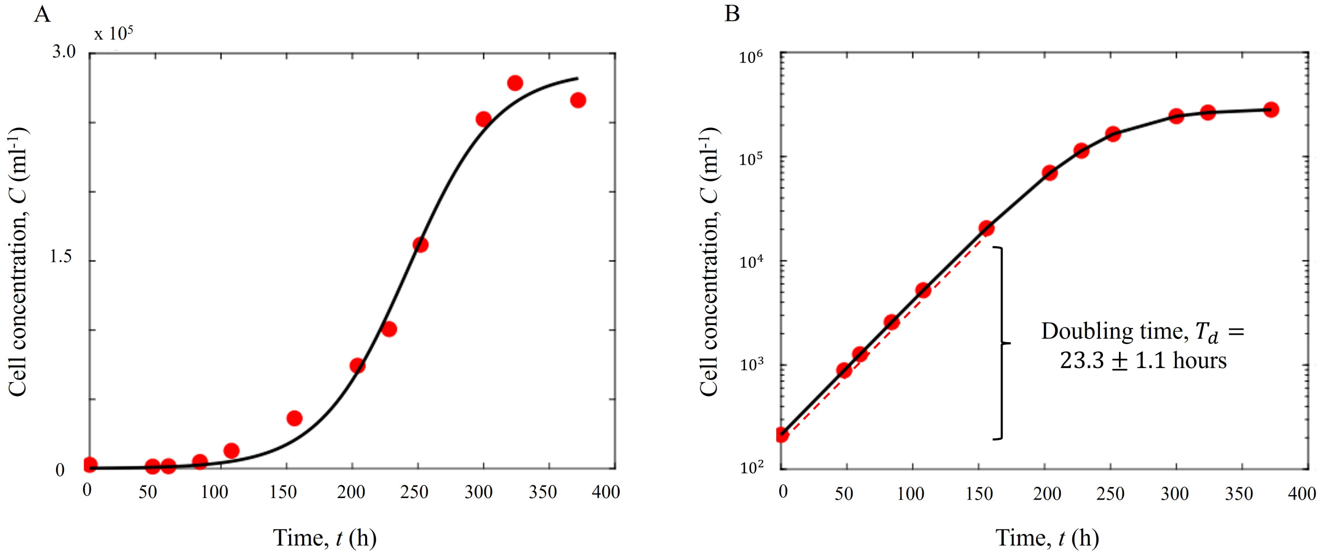

Figure S1. Measuring doubling time based on the population growth curve. (A) Fitting logistic function on cell concentration data, shown here for HA452 population growing under static conditions. Experimental data points are shown as solid red circles, while the corresponding logistic fit is represented by a solid black line. The logistic function was fitted using the least squares error method. (B) To determine the doubling time, the growth curve fitted in (A) was re-plotted on a logarithmic scale, with the solid black line representing the curve and solid red circles indicating the data points derived from the logistic function. The exponential phase of growth was further fitted with an exponential function, shown by a broken red line. The growth rate, obtained from the slope of the straight line (coefficient of the exponential function), was then used to calculate the doubling time, T_d as defined in Materials and Methods Section, consistent with values reported for these cells in the literature (see main text).

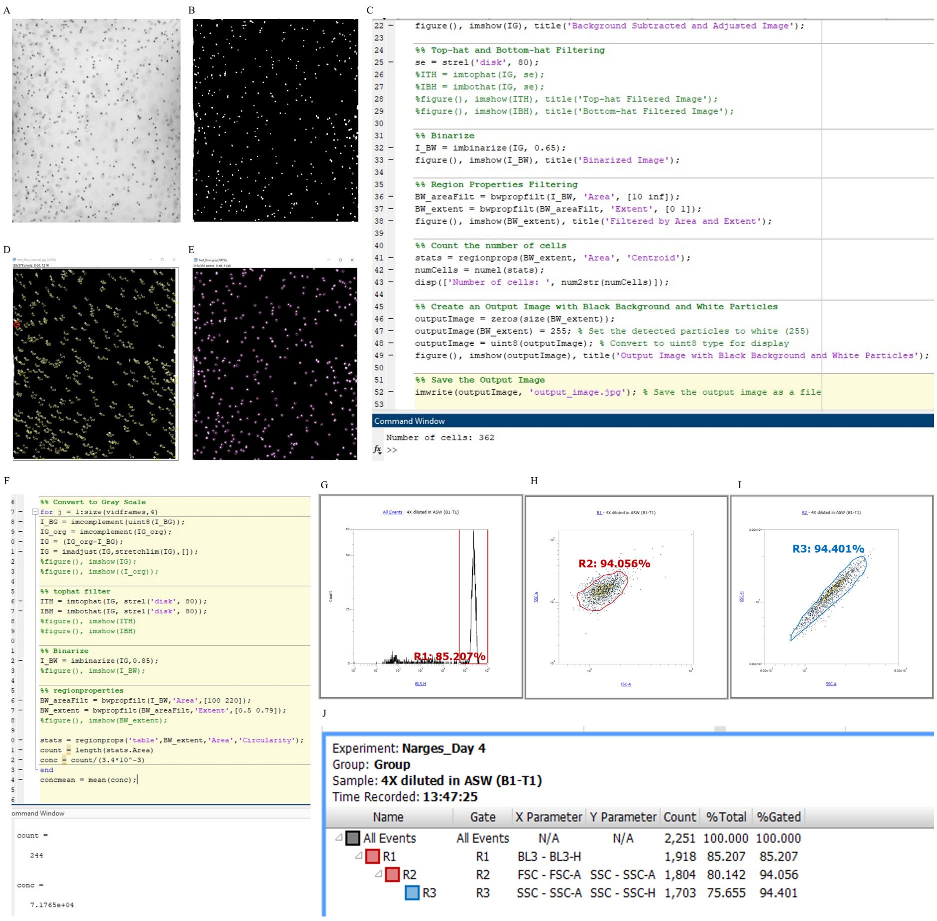

Figure S2. Optimization and validation of codes for cell recognition, quantification, and tracking. (A) Raw images showing the initial view of the chamber containing cells. (B) Processed image showing cells as the bright spots, were obtained by background subtraction and intensity binarization by applying a threshold. 2-step filtering was employed to exclude non-cellular particles: area (100 to 220 pixel^2), and circularity (0.5 to 0.8). (C) Sample coding script for obtaining the cell counts shows a count of 362 cells. (D) Manual cell counting performed on the same frame shown in (A), (B), and (C). The data was validated by manual counting across different frames and experiments; here, the total number of cells was found to be 364, in close agreement with the 362 cells counted by the script. (E) Validation of cell count using the TrackMate algorithm in ImageJ. (F) Cell recognition and count in sequential frames for tracking 160 frames (16 fps over a 10-second duration) for calculating the cell concentration within the test chamber (volume 3.4μl). (G) Flow cytometry histogram obtained from the fluorescence intensity measured in the BL3-H channel (excited by blue light). The gated region was used to identify *H. akashiwo* from debris and non-fluorescent particles. (H) Forward Scatter Area (FSC-A) versus Side Scatter Area (SSC-A) plot of particles gated in R1 based on their auto-fluorescence. These particles, likely phytoplankton, were initially identified by their chlorophyll-induced auto-fluorescence in the BL3-H channel. The tight clustering observed within gate R2 on this scatter plot indicates that the gated population exhibits uniform size and internal complexity, consistent with a homogeneous phytoplankton population. The sequential application of fluorescence gating (R1) followed by scatter gating (R2) enhances the specificity of isolating the phytoplankton, minimizing contamination from debris or non-target particles. (I) Side Scatter Height (SSC-H) versus Side Scatter Area (SSC-A) plot of the cells gated in R2, further refined the population based on granularity. The tight clustering within gate R3, which includes 99.105% of the events from R2, confirms that the identified phytoplankton population is highly homogeneous with consistent granularity, effectively isolating it from non-target particles. (J) Results of the flow cytometry analysis, based on the total number of detected events (cells) within a given sample volume (50 μL). The final concentration matched within 5% of the concentration estimated from the imaging data.

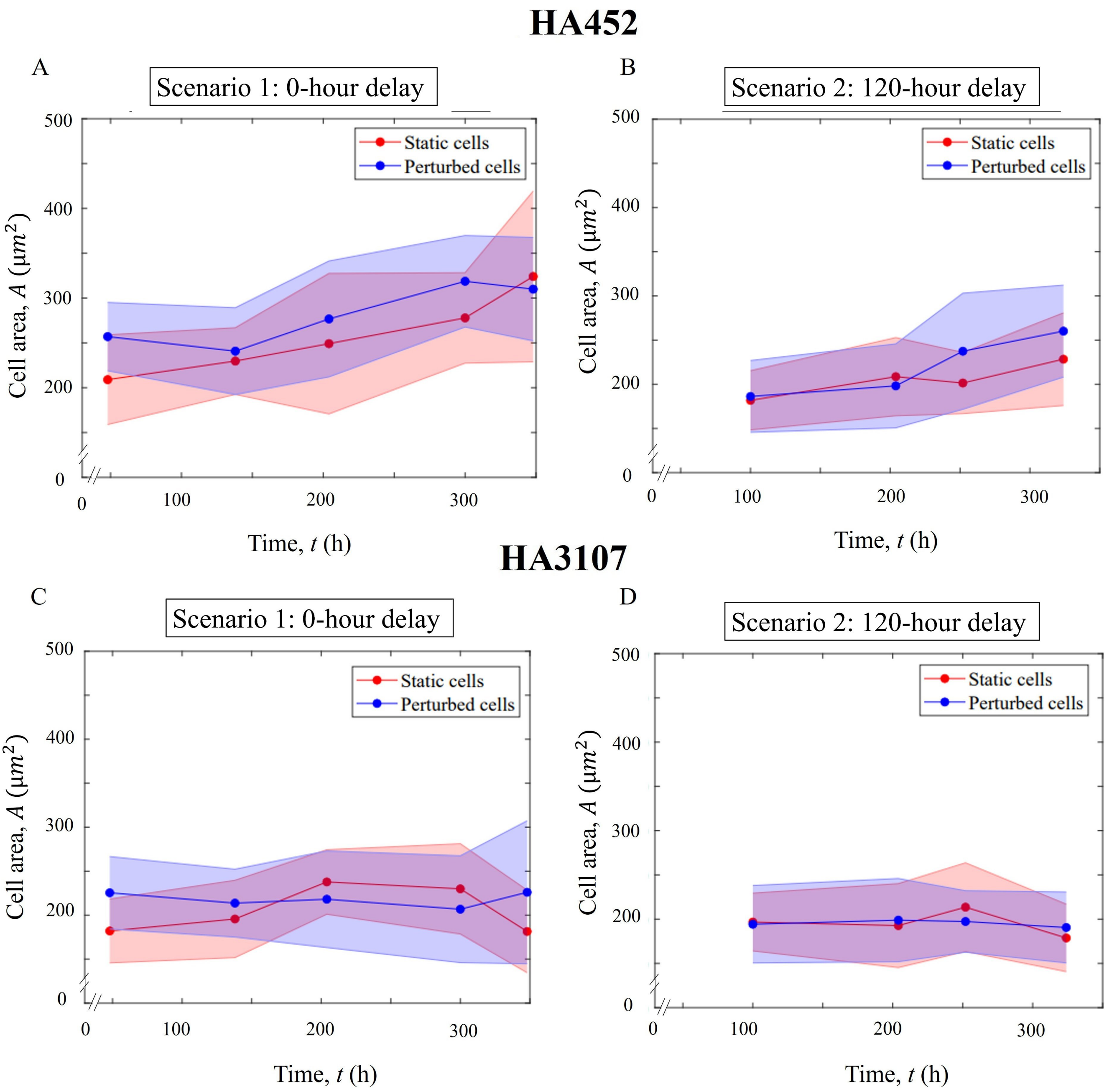

Figure S3. Cell area analysis and representation. (A)–(D): The average cell area of HA452 and HA3107 strains under different delay scenarios, comparing static and perturbed conditions. (A) and (B) show the cell area of HA452 under 0-hour and 120-hour delay scenarios, respectively, while (C) and (D) depict the cell area of HA3107 under the same conditions. The cells analyzed for lipid content were also assessed for cell area. The findings indicate that cell area remained largely uniform between control and perturbed groups for both strains across all scenarios throughout the observation period. For each data point, 20 cells were randomly selected from each biological replicate for analysis. The data represent the mean cell area of all analyzed cells, with the shaded region indicating the standard deviation.

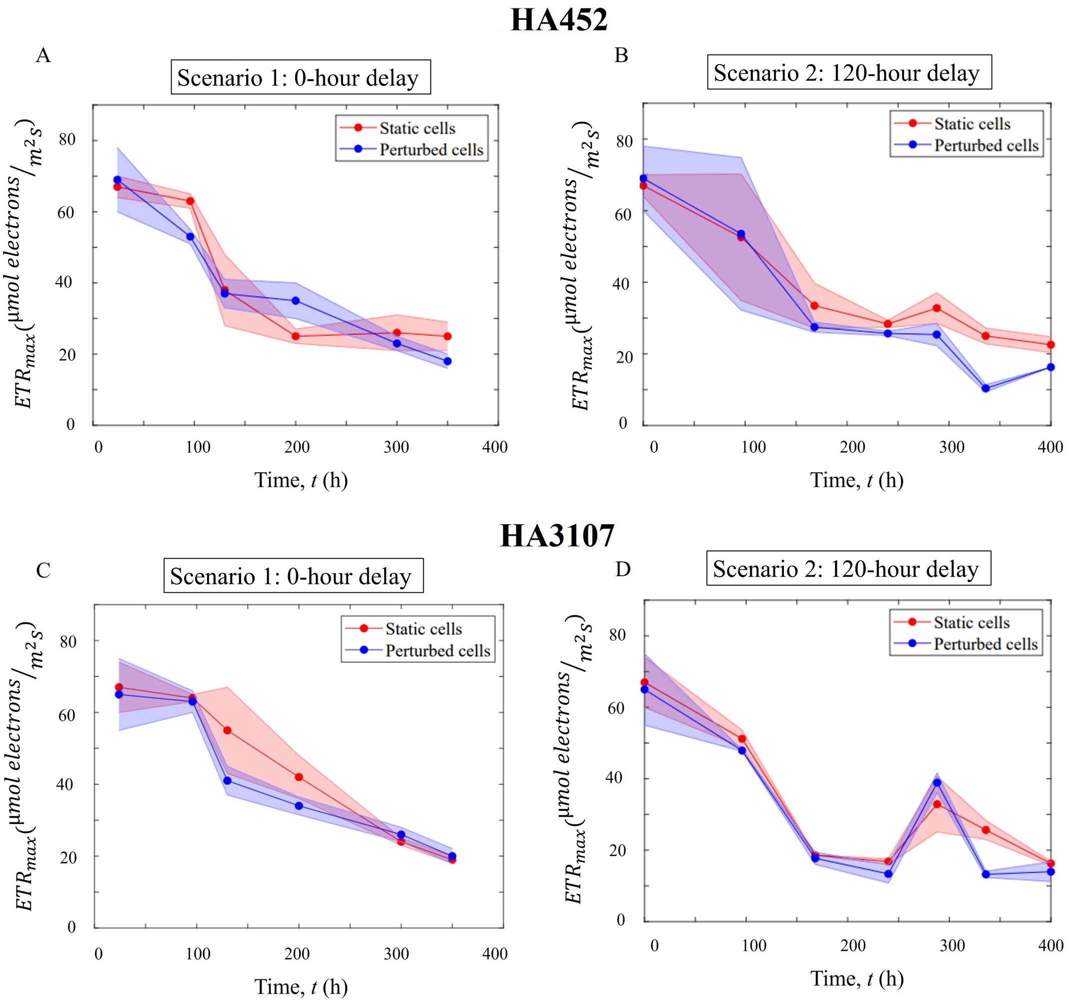

Figure S4. Observed Maximum Electron Transfer Rate (ETR_{max}) values measured across both experimental conditions and all replicates. (A)–(D): The average ETR_{max} of HA452 and HA3107 cells under various delay scenarios, comparing static and perturbed conditions. (A) The ETR_{max} in perturbed HA452 cells under the 0-hour delay setting remains consistent with that of control cells throughout the entire observation period, indicating that both cell groups exhibit similar energy demands from their environment. (B) Under the 120-hour delay scenario, perturbed HA452 cells exhibited a ETR_{max} comparable to that of static cells; however, the ETR_{max} in perturbed cells was consistently lower than in static cells, although this difference was not initially significant. A significant reduction was observed at 250 h, corresponding to 130 h post-perturbation. This suggests that the energy demand in perturbed cells decreased, and, when considered alongside changes in fitness, photosynthetic efficiency, and lipid accumulation, indicates a shift in energy allocation pathways within these cells. (C) The ETR_{max} in perturbed HA3107 cells under the 0-hour delay scenario remained consistent with that of control cells throughout the entire observation period. This indicates that the perturbed cells were able to harvest similar levels of photo energy, sustain comparable rates of photosynthesis, and maintain their overall fitness. (D) Under the 120-hour delay scenario, perturbed HA3107 cells exhibited a ETR_{max} comparable to that of static cells, indicating that the energy demand remained similar between the two groups. However, photosynthetic efficiency decreased significantly immediately following the introduction of the perturbation. For each data point, two technical replicates were obtained from each of the three biological replicates and averaged across all replicates. The shaded region represents the standard deviation.

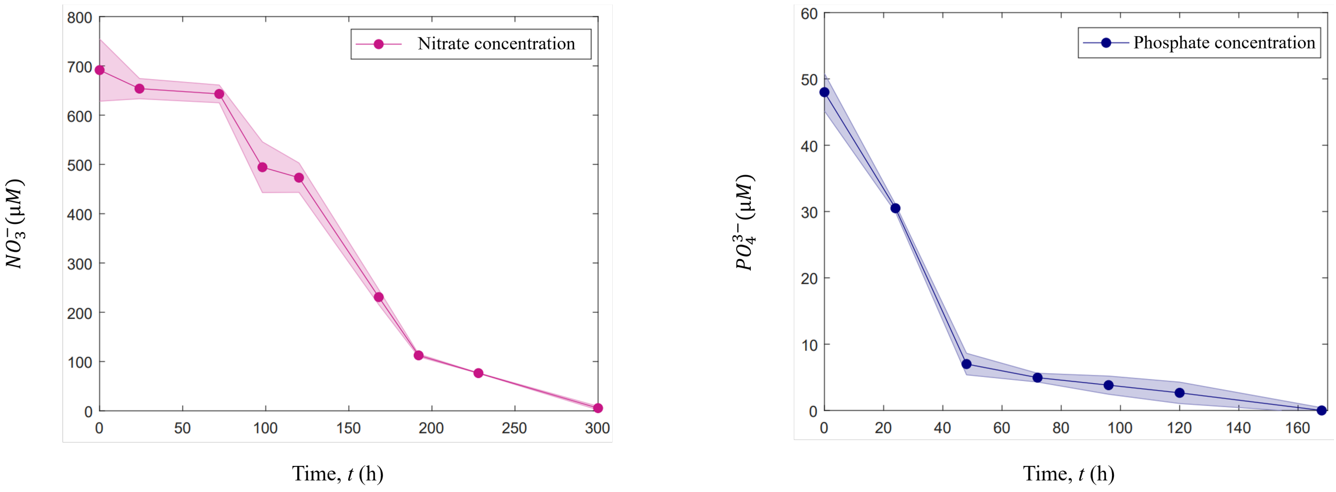

Figure S5. Nitrate and phosphate depletion in cell cultures. (A) Nitrate concentration (NO_3^-) μM over time, shown here for HA3107 cultures grown in nutrient-replete f/2(-Si) medium at 22 °C. Nitrate levels are fully depleted after 300 h of incubation. (B) The concentration of phosphate (PO_4^{3-}) μM over time for HA3107 cultures grown in nutrient-replete f/2(-Si) medium at 22 °C. Phosphate levels are fully depleted after 160 h of incubation. For each data point, two technical replicates were obtained and analyzed for each biological replicate. The shaded region represents the standard deviation calculated from the six resulting measurements, illustrating the variability across the data.
